# A Systematic Computational Method for Practical Identifiability Analysis in Mathematical Models Arising from Biology

**Published:** 2025-01-12

**Authors:** Shun Wang, Wenrui Hao

**Affiliations:** 1 Department of Mathematics, Penn State University, University Park, Pennsylvania, United States of America

**Keywords:** Practical Identifiability, Parameter Regularization, Uncertainty Quantification, Optimal Data Collection

## Abstract

Practical identifiability is a critical concern in data-driven modeling of mathematical systems. In this paper, we propose a novel framework for practical identifiability analysis to evaluate parameter identifiability in mathematical models of biological systems. Starting with a rigorous mathematical definition of practical identifiability, we demonstrate its equivalence to the invertibility of the Fisher Information Matrix. Our framework establishes the relationship between practical identifiability and coordinate identifiability, introducing a novel metric that simplifies and accelerates the evaluation of parameter identifiability compared to the profile likelihood method. Additionally, we introduce new regularization terms to address non-identifiable parameters, enabling uncertainty quantification and improving model reliability. To guide experimental design, we present an optimal data collection algorithm that ensures all model parameters are practically identifiable. Applications to Hill functions, neural networks, and dynamic biological models demonstrate the feasibility and efficiency of the proposed computational framework in uncovering critical biological processes and identifying key observable variables.

## Introduction

In systems biology, mathematical modeling is a widely used and powerful tool for analyzing biological processes across multiple scales. At the microscopic scale, differential equations are used to model intracellular signaling networks^1–3^, including cancer signaling pathways^4^, epithelial-mesenchymal transitions^5^, single-cell RNA velocity^6–8^, and morphogen gradients involved in cell development^9–12^. At the mesoscopic scale, ordinary differential equations (ODEs) are frequently applied to simulate cancer-immune^13–15^ and virus-host immune interactions^16,17^, aiding in the prediction of disease progression. At the macroscopic scale, partial differential equations (PDEs) are employed to describe cell movement and spatial cell-cell interactions, such as tumor cell invasion^18–20^ and spatial interactions of immune cells^21,22^, facilitating predictions of cancer development and cardiovascular disease progression^23–25^.

Due to technical constraints and other limitations, not all parameters in these models can be directly observed. To accurately reflect real-world dynamics, it is essential to calibrate model parameters using observable data. Typically,, the least squares method is employed to estimate unmeasured model parameters based on observable data^26^. However, there may be cases where certain unknown parameters are inherently non-identifiable from the observable data, while others exhibit high sensitivity to it. Such situations can result in different parameter sets producing similar dynamic trajectories, raising significant concerns about the reliability and accuracy of the model’s predictions. Parameter identifiability is, therefore, a critical topic in dynamic systems^27^. In non-identifiable systems, multiple parameter sets can generate identical or similar trajectories from the same dataset, undermining the reliability of predictions and limiting the model’s practical utility.

Parameter identifiability consists of two components: structural identifiability and practical identifiability^28^. Structural identifiability, as prior parameter identifiability, is defined as the condition in which two sets of observed variables or system outputs are identical if and only if their corresponding parameter sets are exactly the same^29^. The primary goal of structural identifiability analysis is to determine whether a model is identifiable by examining its structure before attempting to estimate parameters from data. Several computational methods have been developed for structural identifiability analysis, with differential algebra^29^ and Lie derivatives^30^ being among the most commonly used approaches. Furthermore, various software tools have been designed for structural identifiability analysis of dynamic systems, such as GenSSI2^31^, SIAN^32^, and STIKE-GOLDD^30^. These tools have been benchmarked against standard models to assess and compare their performance^33^. However, structural identifiability analysis relies on two key assumptions: that model structures are entirely accurate and that measurements are error-free^29^. Since these assumptions rarely hold in practice, it is essential to determine whether structurally identifiable parameters can be reliably estimated from noisy data. Therefore, only models that are structural identifiable require further practical identifiability analysis^29^.

Practical identifiability, as posterior parameter identifiability, refers to the ability to assess parameter identifiability based on observed experimental data^29^. Unlike structural identifiability, practical identifiability lacks a rigorous mathematical definition, which remains an urgent issue to be addressed. However, compared to structural identifiability, practical identifiability offers greater potential for application. For instance, one study employed the Hessian matrix to evaluate the practical identifiability of observable and hidden variables in models, enabling the quantification of uncertainties associated with unobservable variables^27^. Additionally, another study utilized non-identifiable parameters to analyze parameter uncertainty when mathematical models were fitted to data^34^. Furthermore, practical identifiability has been applied to design minimally sufficient experiments for pharmacokinetic/pharmacodynamic models that capture the distribution of drugs within the tumor microenvironment^35^. Typically, practical identifiability is evaluated using methods such as calculating the profile likelihood^28,36–38^ or the parameter correlation matrix through the FIM^29,39,40^. However, calculating the profile likelihood is computationally expensive, particularly when the number of model parameters is large. Meanwhile, the FIM-based approach is limited to cases where the FIM is invertible, as all the parameters are practically identifiable if and only if the FIM is invertible^29,39^. Ensuring that model parameters remain practically identifiable when the FIM is singular remains one of the critical challenges to be addressed.

In this paper, we propose a novel and rigorous mathematical definition for practical identifiability, proving that the invertibility of the FIM is a necessary and sufficient condition for all the parameters to be practically identifiable. Using the concept of coordinate identifiability derived from the profile likelihood^36^, we establish the relationship between practical identifiability and coordinate identifiability and introduce a more effective metric for analyzing parameter coordinate identifiability. To address cases where the FIM is singular, we identify the eigenvectors associated with non-identifiable parameters through eigenvalue decomposition (EVD) and incorporate these eigenvectors into practical identifiability and regularization terms, enabling all the parameters to become practically identifiable during model fitting. Additionally, we develop an uncertainty quantification method to assess the influence of non-identifiable parameters on model predictions. Last, we propose a novel algorithm for designing experiments to ensure that the observed data can render all model parameters practically identifiable.

## Results

### Overview of Practical Identifiability Analysis and Its Applications

**Fig. 1.**
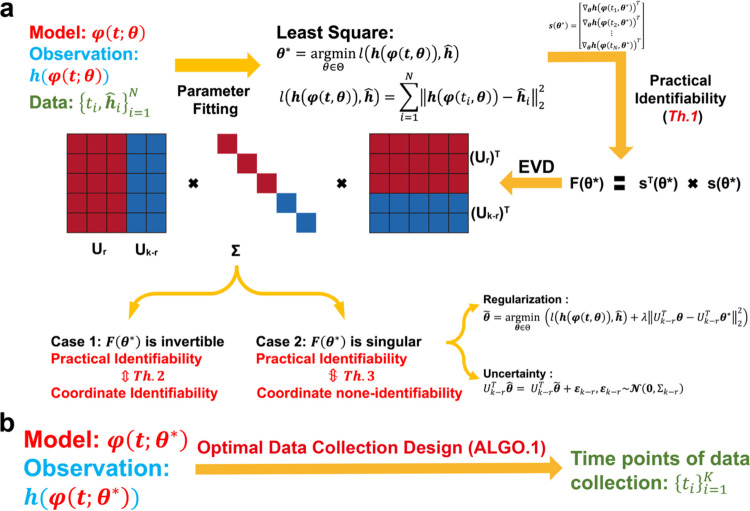
Illustration of the contributions presented in this study. **(a)** A schematic representation of parameter practical identifiability analysis. Practical identifiability is determined by the eigenvalue matrix Σ, which is color-coded: red represents eigenvalues greater than zero, indicating practically identifiable, while blue represents eigenvalues equal to zero, signifying practically non-identifiable. In the eigenvector matrix U, the red portion corresponds to identifiable parameters, $U_{r}^{T}\theta$, while the blue portion corresponds to non-identifiable parameters, $U_{k-r}^{\theta }\theta$. **(b)** The optimization of data collection design informed by practical identifiability.

To systematically conduct practical identifiability analysis for model parameters, we propose a novel and rigorous mathematical definition of practical identifiability (Definition 1 in Materials and Methods). This definition introduces the concept of practical identifiability from a data-fitting perspective, distinguishing it entirely from the concept of structural identifiability. Practical identifiability analysis is based on the specific form of a model φ(t,θ), where t is the variable and $\theta \in R^{k}$ denotes all the parameter. This can refer to any functional form or solution of differential equations, along with observable variables h(φ(t,θ)) and experimental data collected at different time points ${\left\{t_{i},{\hat{h}}_{i}\right\}}_{i=1}^{N}$ as inputs. Then an initial parameter set, $\theta^{*}$, is obtained using a least-squares parameter fitting approach. Next, we calculate a generalized parameter sensitivity matrix $s\left(\theta^{*}\right)$ defined by Eq. (4) to further compute the FIM by $F\left(\theta^{*}\right)=s^{T}\left(\theta^{*}\right)s\left(\theta^{*}\right)$ and perform EVD on it. According to Theorem 1, the parameter θ is practically identifiable if and only if the FIM is invertible. Thus, the practical identifiability is determined by the eigenvalue matrix Σ, where eigenvalues greater than zero indicates the corresponding parameters, $U_{r}^{T}\theta$, are practically identifiable, while eigenvalues equal to zero means the corresponding parameters, $U_{k-r}^{T}\theta$, is practically non-identifiable. This procedure is summarized in Fig. 1a.

We further explore the relationship between practical identifiability and coordinate identifiability (Definition 2 in Materials and Methods) through Theorems 2 and 3 (Details in Materials and Methods). Theorem 2 reveals that coordinate identifiability is equivalent to practical identifiability when the FIM is invertible, while Theorem 3 highlights the differences between coordinate identifiability and practical identifiability when the FIM is singular. Furthermore, we propose the index ${\left\|\left(I-AA^{†}\right)s_{i}\right\|}_{\infty }$ to quantify the identifiability capacity; specifically, the lower the index, the more difficult it is to identify the parameter $\theta_{i}^{*}\in \theta^{*}$ (Details of A and $s_{i}$ are in Theorem3 in Materials and Methods). Moreover, when $F\left(\theta^{*}\right)$ is singular, some parameters are not practically identifiable. Thus, we propose a regularization method based on practical parameter identifiability to ensure that all parameters become practically identifiable during parameter fitting (Fig. 1a, details in ‘Parameter regularization’ section in Materials and Methods). Furthermore, for non-identifiable parameters, we develop a quantitative method to assess the uncertainty they introduce and evaluate their impact on model predictions (Fig. 1a, details in ‘Uncertainty Quantification’ section in Materials and Methods).

Building on the theorems and properties derived from analyzing the practical identifiability of model parameters (Fig. 1a), we propose a novel algorithm for designing experiments to ensure that the observed data renders all model parameters practically identifiable (Fig. 1b, Algorithm 1 in Materials and Methods). Using data or prior empirical information, initial model parameter $\theta^{*}$ can be obtained as inputs to the algorithm. Algorithm 1 then generates a set of time points, representing the moments during the experiment when data measurements should be collected to ensure that all model parameters are practically identifiable.

### Polynomial Fitting Benchmark Example

To evaluate the accuracy of our proposed method, we apply it to a polynomial example as $h(t;\theta )=\theta_{1}+\theta_{2}t^{2}+\theta_{3}[(t-1)(t-2)(t-3)+2]$ (more details are given in Materials and Methods) to compute practical identifiability and compare the results with the profile likelihood method^37^ which serves as a benchmark for practical identifiability analysis.

**Fig. 2.**
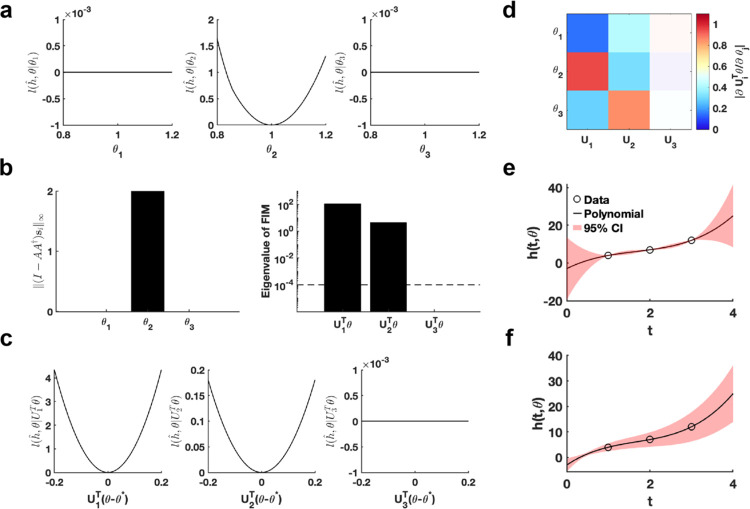
Validation method accuracy in polynomial fitting. **(a)** Coordinate identifiability analysis at $\theta^{*}=[1,1,1{]}^{T}$ using the profile likelihood. **(b)** Two metrics ${\left\|\left(I-AA^{†}\right)s_{i}\right\|}_{\infty }$ and eigenvalue of $F\left(\theta^{*}\right)$ for conducting practical identifiability analysis. The dashed line is the threshold $\varepsilon =10^{-4}$ of eigenvalue of $F\left(\theta^{*}\right)$. **(c)** Coordinate identifiability analysis to parameter $U^{T}\theta^{*}$ using the profile likelihood. **(d)** Heatmap of the eigenvector matrix. The color bar represents the values of each eigenvector element. The shaded area indicates the eigenvectors corresponding to non-identifiable parameters. **(e)** Uncertainty quantification from the perturbation to non-identifiable parameters. **(f)** Uncertainty quantification from the perturbation to all parameters. Circles represent the synthetic data generated from the polynomial function. The solid line represents the polynomial function with the given parameter values $\theta^{*}$. The red area represents the 95% confidence interval under parameter perturbation.

Using the given parameter $\theta^{*}$, we utilize the profile likelihood method^37^ to assess the identifiability of each parameter in the polynomial function, establishing a benchmark for comparison (Fig. 2a). Our proposed method computed two metrics, ${\left\|\left(I-AA^{†}\right)s_{i}\right\|}_{\infty }$ and eigenvalue of $F\left(\theta^{*}\right)$, to evaluate the practical identifiability of the polynomial function parameters. The result of ${\left\|\left(I-AA^{†}\right)s_{i}\right\|}_{\infty }$ demonstrates that only parameter $\theta_{2}$ is identifiable, whereas $\theta_{1}$ and $\theta_{3}$ are non-identifiable (Fig. 2b), consistent with the benchmark results (Fig. 2a). The eigenvalue of $F\left(\theta^{*}\right)$ further reveals the practical identifiability. Specifically, $U_{1}^{T}\theta$ and $U_{2}^{T}\theta$ are identifiable while the parameter $U_{3}^{T}\theta$ is non-identifiable, as shown in Fig. 2b. To emphasize the discrepancy with the profile likelihood method, we perform a linear transformation on the parameters, namely, $U^{T}\left(\theta -\theta^{*}\right)$. Then we conduct further practical identifiability analysis on the parameters using the profile likelihood method, which indicates that parameters $U_{1}^{T}\theta$ and $U_{2}^{T}\theta$ are identifiable, whereas parameter $U_{3}^{T}\theta$ is non-identifiable (Fig. 2c). These results align perfectly with the parameter identifiability analysis shown in Fig. 2b but shows the sensitivity of the profile likelihood method. Leveraging this matrix U, we incorporate a regularization term into the loss function to ensure that each parameter achieves practical identifiability. Subsequently, we perform the profile likelihood method to assess the identifiability of each parameter in the regularized loss function. The results confirm that all parameters become identifiable following the inclusion of the regularization term (Fig. S1 in Supplementary Materials).

Finally, we introduce parameter perturbations and calculate the 95% confidence interval for variations in the dependent variable. As shown in Fig. 2e, the confidence interval is nearly zero at the data points, indicating that the loss function remains unaffected by perturbations to non-identifiable parameter only. Conversely, the result presented in Fig. 2f shows that the perturbations to all parameter influence the loss function at the data points, confirming that the loss function changes in response to perturbations to all parameter. This result highlights that our proposed uncertainty quantification method more precisely captures the prediction errors arising from parameter uncertainty. This accuracy is achieved because our method maintains the loss function’s minimum under parameter perturbations.

### Hill Functions and Neural Networks

Next, we perform our proposed parameter practical identifiability analysis method to Hill functions and neural network functions, two widely used nonlinear models in systems biology. The primary objective is to determine whether our method can uncover the biological insights underlying these nonlinear functional classes.

**Fig. 3.**
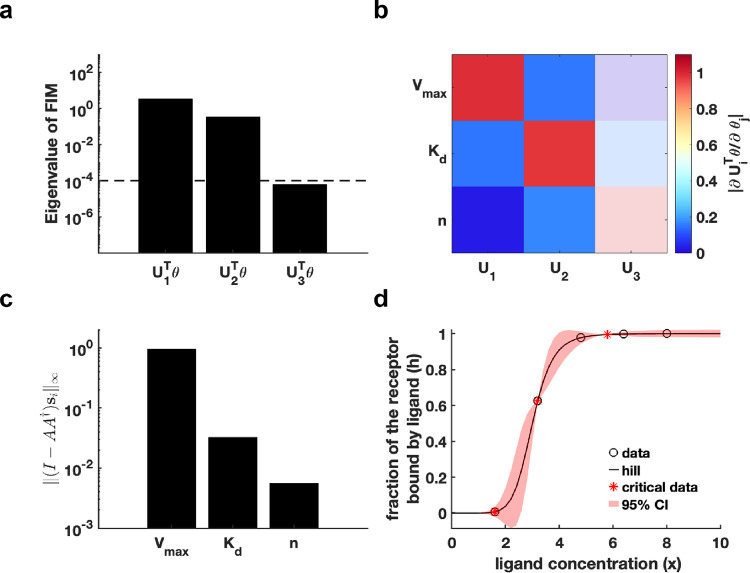
Practical identifiability analysis to Hill function. **(a)** Eigenvalue of $F\left(\theta^{*}\right)$. The dashed line is the threshold $\varepsilon =10^{-4}$ of eigenvalue of $F\left(\theta^{*}\right)$. **(b)** Heatmap of the eigenvector matrix. The color bar represents the values of each eigenvector element. The shaded area indicates the eigenvectors corresponding to non-identifiable parameters. **(c)** Coordinate identifiability analysis to parameter $\theta^{*}$ using the metric ${\left\|\left(I-AA^{†}\right)s_{i}\right\|}_{\infty }$. **(d)** Uncertainty quantification from the perturbation to none-identifiable parameters. Circles represent the synthetic data generated from the Hill function. The solid line represents the Hill function with the given parameter values $\theta^{*}$. The red area represents the 95% confidence interval under parameter perturbation. The star represents the critical data identified by algorithm 1 (Details in Materials and Methods).

First, we generated a synthetic dataset using predefined parameter $\theta^{*}$ for Hill function $h(t;\theta )=V_{max}\frac{x^{n}}{x^{n}+K_{d}^{n}}$ (Details in Materials and Methods). We establish an eigenvalue threshold $\varepsilon =10^{-4}$ to classify eigenvectors corresponding to eigenvalues below this threshold as non-identifiable parameters. The analysis shown in Fig. 3a reveals that parameter $U_{3}^{T}\theta$ is non-identifiable, while parameters $U_{1}^{T}\theta$ and $U_{2}^{T}\theta$ are practically identifiable. Examination of the eigenvector matrix further confirms that parameters $V_{max}$ and $K_{d}$ are identifiable, whereas the parameter n is non-identifiable (Fig. 3b). Using metric ${\left\|\left(I-AA^{†}\right)s_{i}\right\|}_{\infty }$, we determine that parameter $V_{max}$ exhibits the highest practical identifiability, followed by parameter $K_{d}$, with parameter n showing the lowest identifiability (Fig. 3c). Furthermore, we employ the profile likelihood method as a benchmark for analyzing the identifiability of Hill function parameters, yielding results that were fully consistent with those derived from our proposed method (Fig. S2a in Supplementary Materials). Our practical identifiability analysis reflects that parameter n in the Hill function requires prior biological information for determination, as data fitting alone is insufficient to reliably identify its exact value. Finally, we also introduce parameter perturbations and calculate the 95% confidence interval for variations in the dependent variable. The results in Fig. 3d demonstrate that perturbations to the non-identifiable parameter n primarily impact the region near the inflection point of the Hill equation. Compared to the confidence interval derived from perturbing all parameters (Fig. S2b in Supplementary Materials), the findings in Fig. 3d more closely reflect the actual data-fitting process of the Hill function. Moreover, we utilize Algorithm 1 to determine the critical time points of the Hill function, identifying the time points that render all parameters practically identifiable (Fig. 3d).

**Fig. 4.**
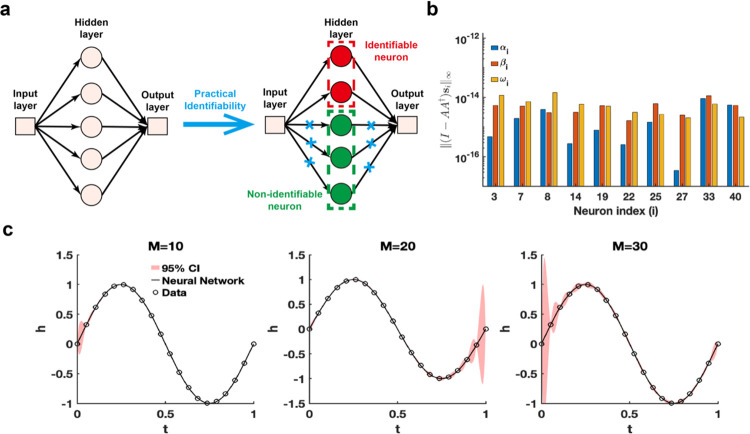
Practical identifiability analysis of neural network with one hidden layer. **(a)**. Schematic of parameter practical identifiability applied in neural network. **(b)**. Identifiable neurons recognized by the metric $\|(I-\left.AA^{†}\right)s_{i}{\|}_{\infty }$ when the activation function set to the ReLu function and the number of neurons is assigned as 40. **(c)**. Uncertainty quantification was performed by introducing perturbations to non-identifiable parameters across different numbers of neurons (M), with the activation function set to the tanh function. Circles represent the synthetic data generated from the sine function sin(2πt), t∈[0,1]. The solid line represents the neural network with the given parameter values $\theta^{*}$. The red area represents the 95% confidence interval under non-identifiable parameter perturbation.

The single hidden-layer neural network is constructed to fit a sine function sin(2πt), t∈[0,1], leveraging parameter practical identifiability analysis to pinpoint neurons with practically identifiable parameters. For neurons deemed non-identifiable, regularization terms are introduced to fix their parameters during training, allowing the model to concentrate exclusively on training the parameters of identifiable neurons (Fig. 4a). This approach holds promise for expediting the training process and improving prediction accuracy. When the activation function set to the ReLu function and the number of neurons is assigned as 40, we utilize the metric ${\left\|\left(I-AA^{†}\right)s_{i}\right\|}_{\infty }$ to recognize the identifiable neurons (Fig. 4b). The remaining neurons are classified as non-identifiable because their metrics ${\left\|\left(I-AA^{†}\right)s_{i}\right\|}_{\infty }$ are presented as zero. When the activation function is changed as the tanh function, the metrics ${\left\|\left(I-AA^{†}\right)s_{i}\right\|}_{\infty }$ for all neurons are positive (Fig. S3a in Supplementary Materials). Furthermore, the 95% confidence intervals are computed for varying numbers of neurons, revealing that uncertainty increases as the number of neurons grows (Fig. 3c). The result of eigenvalue distribution across varying numbers of neurons (Fig. S3b in Supplementary Materials) shows that the ratio of eigenvalues exceeding the threshold decreases as the number of neurons increases. The findings presented indicate that an excessive number of neurons in a single-layer neural network heightens parameter-induced uncertainty, potentially slowing down the training process and increasing the risk of the Runge phenomenon.

### Various Biological Systems with differential equations.

#### LV model.

**Fig. 5.**
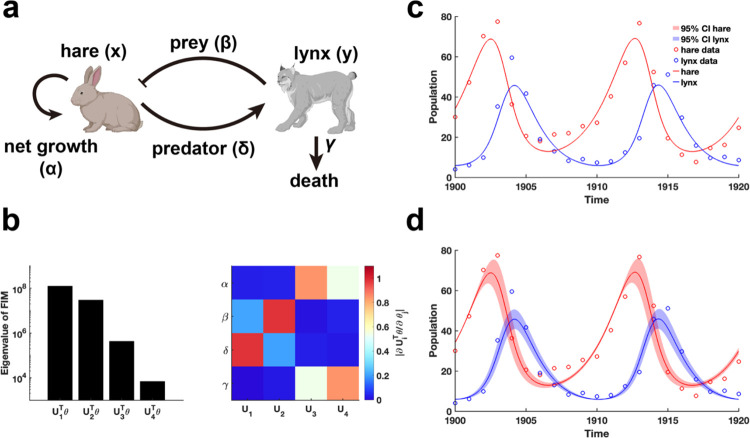
Practical identifiability analysis of LV model. **(a)**. Schematic of LV model. **(b)**. Eigenvalue of $F\left(\theta^{*}\right)$ and heatmap of the eigenvector matrix. The color bar represents the values of each eigenvector element. The parameter $\theta^{*}$ values are provided in the “Parameter Values“ section of the Supplementary Materials. **(c)**. Uncertainty quantification is performed by introducing perturbations to non-identifiable parameters. **(d)** Uncertainty quantification from the perturbation to all parameters. Circles represent the real data of hare and lynx obtained from published literature^42^. The solid line represents the LV model with the given parameter values $\theta^{*}$. The red area represents the 95% confidence interval under parameter perturbation.

We begin by examining the classic predator-prey relationship within ecological network models using the LV model^41^ (Fig. 5a). Public data on hare and lynx populations^42^ are utilized for parameter estimation through data fitting. Using the obtained parameters $\theta^{*}$, we calculate the FIM $F\left(\theta^{*}\right)$ and conduct EDV to derive the eigenvalues and their corresponding eigenvectors (Fig.5b). Our analysis reveals that parameters (β,δ) associated with the predator-prey interaction exhibited the highest eigenvalues, followed by the intrinsic growth and death rates of the species (Fig. 5b). This finding indicates that the periodic fluctuations observed in the hare and lynx populations are predominantly driven by the predator-prey interaction parameters, emphasizing their role in inducing periodic dynamics. Moreover, the invertibility of the FIM confirm that the parameters are uniquely identifiable without uncertainty (Fig. 5c and **Theorem 1**). Although the confidence intervals derived from perturbing all parameters exhibit periodic variations (Fig. 5d), the perturbed parameters failed to preserve the loss function in data fitting.

#### Michaelis–Menten system.

**Fig. 6.**
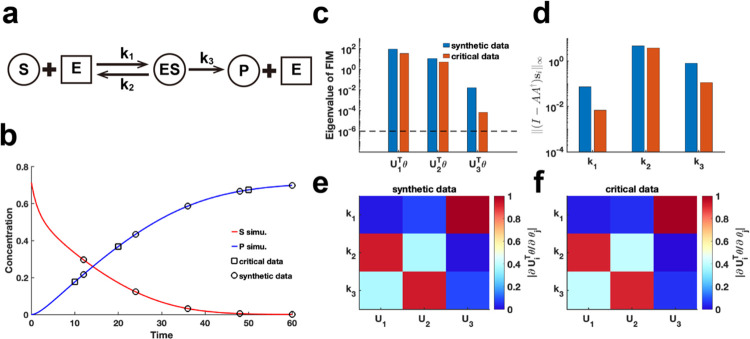
Practical identifiability analysis of Michaelis–Menten system. **(a)**. Schematic of Michaelis–Menten system. S, E, ES, and P represent substrate, enzymes, complex of substrate and enzymes, and product, respectively. **(b)**. Time course of substrate and product. Circles represent the synthetic data generated by the given parameter values $\theta^{*}$^44^. The solid line represents the Michaelis–Menten model with the given parameter values $\theta^{*}$. Squares represent the critical data identified by algorithm 1. **(c)**. Eigenvalue of $F\left(\theta^{*}\right)$ using the synthetic data and critical data respectively. The dash line is the threshold $\varepsilon =10^{-6}$. **(d)**. Coordinate identifiability analysis to parameter $\theta^{*}$ using the metric ${\left\|\left(I-AA^{†}\right)s_{i}\right\|}_{\infty }$ for the synthetic data and critical data. **(e)**. Heatmap of the eigenvector matrix using synthetic data. **(f)**. Heatmap of the eigenvector matrix using synthetic data. The color bar represents the values of each eigenvector element. The parameter $\theta^{*}$ values are provided in the “Parameter Values“ section of the Supplementary Materials.

We extend our method to assess the practical identifiability of parameters in the classic enzyme-catalyzed reaction model, the Michaelis–Menten system^43^ (Fig. 6a). Using parameters $\theta^{*}$ obtained from the literature^44^, we generate synthetic data with the observable variable set as the substrate and product concentration (Fig. 6b) (case (1) of Michaelis–Menten system in Materials and Methods). Additionally, we alter the observable variable to the product concentration (case (2) of Michaelis–Menten system in Materials and Methods) and perform Algorithm 1 to identify critical data points. By comparing the eigenvalue distributions of the FIM $F\left(\theta^{*}\right)$ generated using synthetic data and critical data, we consistently observe that parameter $k_{1}$ exhibited the lowest identifiability (Figs. 6c, e, f). Using our proposed metric ${\left\|\left(I-AA^{†}\right)s_{i}\right\|}_{\infty }$ to analyze coordinate identifiability, we confirm that $k_{1}$ has the lowest identifiability among the parameters. This finding highlights the difficulty in accurately capturing the chemical reaction constant associated with the binding of the enzyme to the substrate, regardless of whether substrate or product data are utilized. Moreover, the eigenvector matrices derived from both data types are identical (Figs. 6e–f), indicating that Algorithm 1 effectively guides experimental design for optimizing data measurement in the Michaelis–Menten system.

#### SEIR model.

**Fig. 7.**
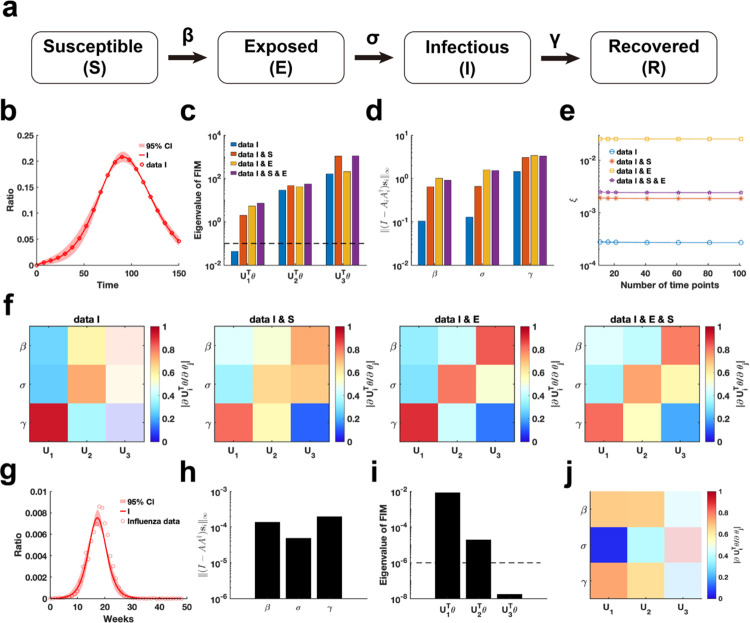
Practical identifiability analysis of SEIR model. **(a)**. Schematic of SEIR model. **(b)**. Uncertainty quantification is performed by introducing perturbations to non-identifiable parameters. Circles represent the synthetic data of Infected ratio when the parameter values are given. The solid line represents the infected ratio of SEIR model with the given parameter values. The red area represents the 95% confidence interval under parameter perturbation. **(c)**. Eigenvalue of FIM in the four cases of observable variables (Details in Materials and Methods). The dash line is the threshold $\varepsilon =10^{-6}$. **(d)**. Coordinate identifiability analysis to parameter using the metric ${\left\|\left(I-AA^{†}\right)s_{i}\right\|}_{\infty }$ for four cases of observable variables. **(e)**. Contribution of different data types to parameter practical identifiability using the metric ξ across multiple time points. **(f)**. Heatmap of the eigenvector matrix in the four cases of observable variables. **(g)**. Uncertainty quantification is performed by introducing perturbations to non-identifiable parameters. Circles represent the influenza data of Infected ratio obtained from the CDC website. The solid line represents the infected ratio of SEIR model with the given parameter values. The red area represents the 95% confidence interval under parameter perturbation. **(h)**. Coordinate identifiability analysis to parameter using the metric ${\left\|\left(I-AA^{†}\right)s_{i}\right\|}_{\infty }$ for the influenza data. **(i)**. Eigenvalue of FIM using the influenza data. The dash line is the threshold $\varepsilon =10^{-6}$. **(j)**. Heatmap of the eigenvector matrix using the influenza data. The color bar represents the values of each eigenvector element. The parameter values are provided in the “Parameter Values“ section of the Supplementary Materials.

We employ our proposed parameter practical identifiability method to investigate the SEIR infectious disease model^45^, a system distinguished by its greater number of state variables compared to parameters (Fig. 7a). First, we utilized synthetic data to evaluate the practical identifiability of the model parameters. With the observable variable designated as h(t,θ)=I(t,θ), synthetic data are generated using a specific parameter $\theta^{*}$ (See in the “Parameter Values“ section of the Supplementary Materials). Parameter uncertainty analysis based on the synthetic data indicated that the uncertainty in infected patient data is notably higher during the early stages of the outbreak (Fig. 7b). In contrast, uncertainty analysis conducted by perturbing all parameters demonstrates nearly zero uncertainty in the infected patient data (Fig. S3 in Supplementary Materials).

Next, we analyze the eigenvalue distributions of the FIM (Fig. 7c) and evaluate the coordinate identifiability of parameters across four different scenarios of observable variables using the metric ${\left\|\left(I-AA^{†}\right)s_{i}\right\|}_{\infty }$ (Fig. 7d). Our findings demonstrate that increasing the number of observable variables ensures that all parameters become practically identifiable and significantly enhances the identifiability of each parameter in the model. When the observable variable is set to h(t,θ)=[E(t,θ),I(t,θ)], the data provides the highest contribution to parameter identifiability within the model (Fig. 7d). This suggests that, in the SEIR model, focusing on monitoring exposed and infected individuals is sufficient for accurately predicting the later stages of an epidemic. Additionally, a comparison of the eigenvector matrices showed that, with h(t,θ)=[E(t,θ),I(t,θ)] as the observable variable, the weight of each eigenvector is concentrated on a single parameter. This result underscores the importance of monitoring exposed and infected individuals, as it maximizes the identifiability of individual parameters within the SEIR model.

Finally, we estimated the parameters of the SEIR model using influenza A data from the 2004–2005 season, obtained from the CDC website (Details in Data Availability), and analyzed the practical identifiability of the estimated parameters. Uncertainty analysis revealed that the model predictions exhibit the highest levels of uncertainty during the initial stages and at the peak of the influenza outbreak. Using metric ${\left\|\left(I-AA^{†}\right)s_{i}\right\|}_{\infty }$, it shows that the identifiability of transmission rate (β) and the recovery rate (γ) is nearly identical, while the incubation rate (σ) exhibits the lowest identifiability. The eigenvalue distribution of the FIM and the corresponding eigenvector matrix further confirm the low identifiability of the incubation rate (σ). These findings underscore the critical importance of monitoring exposed individuals to enhance the predictive accuracy of the SEIR model.

#### Cascade model of Alzheimer’s Disease.

**Fig. 8.**
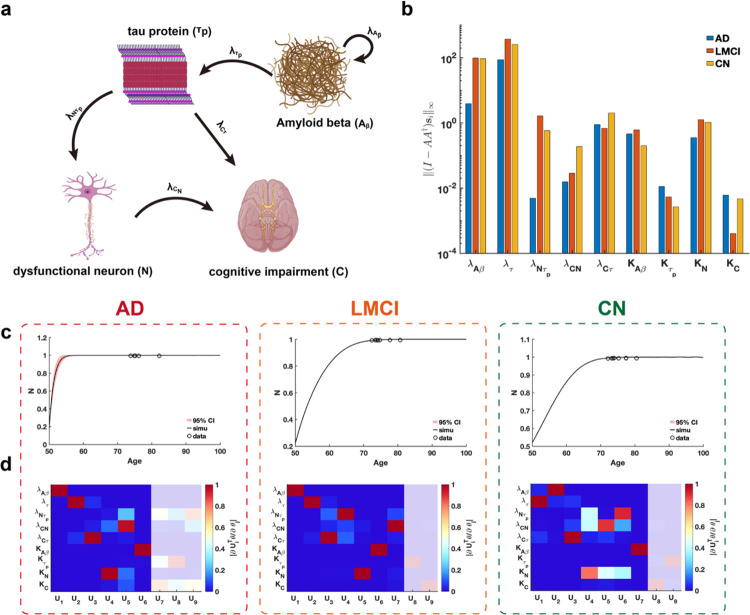
Practical identifiability analysis to cascade model of Alzheimer’s Disease. **(a)**. Schematic of cascade model of Alzheimer’s Disease. **(b)**. Coordinate identifiability analysis to parameter using the metric ${\left\|\left(I-AA^{†}\right)s_{i}\right\|}_{\infty }$. **(c)**. Uncertainty quantification of dysfunctional neuron (N) is performed by introducing perturbations to non-identifiable parameters. Circles represent the real data of AD, LMCI and CN patients. The solid line represents the time course of dysfunctional neuron with the given parameter values. The red area represents the 95% confidence interval under parameter perturbation. **(d)**. Heatmap of the eigenvector matrix using the real data of AD, LMCI and CN patients. The shaded area indicates the eigenvectors corresponding to non-identifiable parameters. The color bar represents the values of each eigenvector element. All parameter values are provided in the “Parameter Values“ section of the Supplementary Materials.

We conduct a practical parameter identifiability analysis on biomarker cascade model of Alzheimer’s Disease (AD)^46^, incorporating data from three clinical groups: cognitively normal (CN), late mild cognitive impairment (LMCI), and AD (Fig. 8a) from Alzheimer’s Disease Neuroimaging Initiative(ADNI) dataset. The primary goal is to leverage practical identifiability analysis to identify variations in model parameters across these groups, thereby uncovering critical biological processes that distinguish the clinical conditions.

Using metric ${\left\|\left(I-AA^{†}\right)s_{i}\right\|}_{\infty }$, we assess the identifiability of each parameter across the three clinical symptom groups and identify two key parameters, growth rate of $\text{N}\left(\lambda_{N\tau_{p}}\right)$ and carrying capacity of $\text{C}\left(K_{C}\right)$, that significantly distinguish these groups (Fig. 8b). Our analysis revealed that parameter $\lambda_{N\tau_{p}}$ demonstrates substantially lower identifiability in AD patients compared to CN and LMCI groups, whereas parameter $K_{C}$ exhibits markedly reduced identifiability in LMCI patients relative to the others (Fig. 8b). These findings suggest that evaluating the identifiability of parameters $\lambda_{N\tau_{p}}$ and $K_{C}$ within the cascade model provides a robust means of distinguishing between CN, LMCI, and AD patients.

Using the eigenvalues and eigenvectors of the FIM for practical identifiability analysis across the three patient groups, we identify that uncertainty in early predictions of dysfunctional neurons is uniquely observed in AD patients (Fig. 8c), whereas predictions for other biomarkers were consistently well-determined across all groups (Figs. S5a, c, e in Supplementary Materials). By establishing a threshold for the eigenvalues (Figs. S5b, d, f in Supplementary Materials)., we observe that AD patients exhibit a greater number of non-identifiable parameters compared to LMCI and CN groups. These findings imply that, given comparable data types and quantities, patients with a higher proportion of non-identifiable parameters identified through FIM analysis are more likely to be diagnosed with AD.

#### Partial differential equation (PDE) model of cancer-immune interactions.

**Fig. 9.**
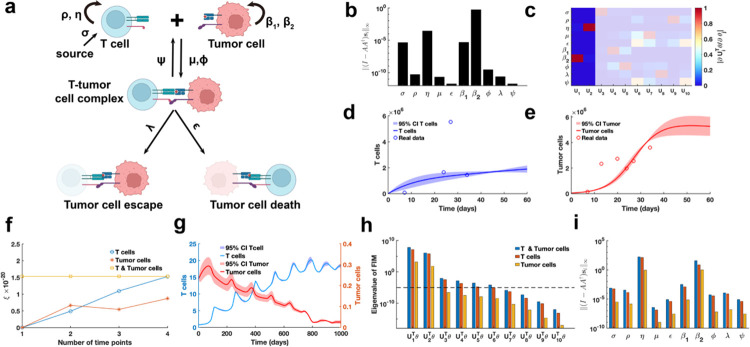
Practical identifiability analysis to PDE model of cancer-immune interactions. **(a)**. Schematic of PDE model of cancer-immune interactions. **(b)**. Coordinate identifiability analysis to parameter using the metric $\|(I-\left.AA^{†}\right)s_{i}{\|}_{\infty }$ based on glioblastoma data. **(c)**. Heatmap of the eigenvector matrix using the glioblastoma data. The shaded area indicates the eigenvectors corresponding to non-identifiable parameters. The color bar represents the values of each eigenvector element. **(d)**. Uncertainty quantification of T cells is performed by introducing perturbations to non-identifiable parameters. Circles represent the experimental data of T cells. The solid line represents the time course of T cells with the given parameter values. The red area represents the 95% confidence interval under parameter perturbation. **(e)**. Uncertainty quantification of tumor cells is performed by introducing perturbations to non-identifiable parameters. Circles represent the experimental data of tumor cells. The solid line represents the time course of tumor cells with the given parameter values. The red area represents the 95% confidence interval under parameter perturbation. **(f)**. Contribution of different data types to parameter practical identifiability using the metric ξ across multiple time points. **(g)**. Uncertainty quantification of both T and tumor cells from the non-identifiable parameters. **(h)**. Eigenvalue of FIM in the three cases of observable variables (Details in Materials and Methods). The dash line is the threshold $\varepsilon =10^{-5}$. **(i)**. Coordinate identifiability analysis to parameter using the metric ${\left\|\left(I-AA^{†}\right)s_{i}\right\|}_{\infty }$ for three cases of observable variables.

As the final example, our proposed parameter identifiability method is employed to investigate the classic cancer-immune interaction PDE model (Fig. 9a)^47^. In contrast to above analyses of biological system models, this model accounts for stochastic cell movement and intricate interaction mechanisms (Fig. 9a), thereby increasing the complexity of the parameter practical identifiability analysis. Our aim is to leverage practical identifiability analysis to uncover critical biological processes of cancer-immune interactions embedded in the model and to determine the key observable variables.

Using public glioblastoma data^14^, which included multiple time-point measurements of T cells and tumor cells, we estimated the parameters of the cancer-immune interaction PDE model, except for the tumor cell random movement parameter ω, which was determined based on prior information. $h(t,\theta )=\left[\int_{0}^{1}E(t,x;\theta )dx,\int_{0}^{1}T(t,x;\theta )dx\right]$ is observable variable for the glioblastoma data. Using metric ${\left\|\left(I-AA^{†}\right)s_{i}\right\|}_{\infty }$, we observe that parameters $\left(\sigma ,\eta ,\beta_{1},\beta_{2}\right)$ with high identifiability are predominantly associated with the biological processes of T cell and tumor cell proliferation and apoptosis (Fig. 9b). In contrast, parameters (μ,ϵ,ϕ,λ,ψ) linked to T cell-tumor cell interactions exhibit low identifiability. Based on the identifiability threshold (Fig. S6a in Supplementary Materials), we identify that the most identifiable parameters are those related to T cell and tumor cell proliferation (Fig. 9c). Uncertainty quantification for T cells presents the high levels of uncertainty in their counts during the early stages of the process (Fig. 9d). Conversely, for tumor cells, the high uncertainty is observed in the later stages, where their counts stabilized at a steady-state level (Fig. 9e).

To investigate the influence of spatial cell movement on parameter practical identifiability, we generate synthetic data with spatial information based on predefined parameters. In this context, three scenarios of observable variables are analyzed (Details in Materials and Methods). Using metric ξ, we evaluate the contributions of these variables to the identifiability of model parameters and find that T cell data provides greater contributions to parameter identifiability compared to tumor cell data (Fig. 9f). Uncertainty quantification using the synthetic data reveals that tumor cell counts exhibits greater uncertainty during the early stages (Fig. 9g). Analysis of the eigenvalue distribution of the FIM (Fig. 9h) and its corresponding eigenvector matrix (Fig. S6b) shows that T cell data allows more parameters in the model to be practically identifiable compared to tumor cell data. Metric ${\left\|\left(I-AA^{†}\right)s_{i}\right\|}_{\infty }$ further demonstrates that T cell data renders parameters (μ,ϵ,ϕ,λ,ψ) related to T cell-tumor cell interactions practically identifiable, whereas tumor cell data primarily identifies parameters $\left(\sigma ,\eta ,\beta_{1},\beta_{2}\right)$ associated with the proliferation of T cells and tumor cells (Fig. 9i). These results underscore the importance of prioritizing the collection of T cell data in practical experiments to improve the model’s capacity for accurately predicting cancer progression.

## Discussions

Practical identifiability is a fundamental aspect of mathematical modeling in biological systems, as it directly influences the reliability and robustness of model predictions. However, despite its significance, it is frequently underappreciated in modeling studies. In this paper, we propose a novel framework for practical identifiability analysis that integrates the concept of coordinate identifiability. Additionally, we introduce an optimal data collection algorithm that utilizes practical identifiability to guide experimental design, thereby improving the efficiency and precision of data acquisition.

We introduce a rigorous mathematical definition for practical identifiability (Definition 1 in Materials and Methods). While the invertibility of the FIM has often been used as a criterion for practical identifiability^27,29,39^, its theoretical foundation has remained unproven. Building on our proposed definition, we formally establish the relationship between parameter practical identifiability and the invertibility of the FIM (Theorem 1 in Materials and Methods). Additionally, we elucidate the relationship between practical identifiability and structural identifiability (Theorem 4 in Materials and Methods), which reveals that if the parameter $\theta^{*}$ is structurally identifiable, we can discover the time series ${\left\{t_{i_{j}}\right\}}_{j=1}^{M}$ to make $\theta^{*}$ practically identifiable.

Coordinate identifiability has received considerable attention in the analysis of dynamic models within systems biology. Traditionally, the profile likelihood method has been used to evaluate the identifiability of individual parameters^28,36,37^. However, this method becomes computationally infeasible for high-dimensional models, such as the cell cycle signaling pathway model with 48 parameters^48^, posing challenges for accurately assessing parameter identifiability. First, we establish that practical identifiability and coordinate identifiability are equivalent when the FIM is invertible (Theorem 2 in Materials and Methods). Second, for cases where the FIM is singular, we introduce a novel metric ${\left\|\left(I-AA^{†}\right)s_{i}\right\|}_{\infty }$ to evaluate the identifiability of individual parameters. We further demonstrate that this metric acts as a linear approximation to the profile likelihood method (Theorem 3 in Materials and Methods). Compared to the profile likelihood approach, our proposed method significantly reduces computational cost while offering a more precise analysis of parameter identifiability.

For cases where the FIM is singular, we approach the problem from two perspectives: introducing regularization terms and refining parameter uncertainty quantification. First, in systems biology, previous studies have utilized regularization techniques, such as Tikhonov regularization or functions derived from prior information, to constrain specific parameters during optimization, effectively preventing changes in the loss function^26,49,50^. Expanding on this concept, we propose a novel regularization term based on parameter practical identifiability analysis (Fig. 1). Our approach targets non-identifiable parameters by incorporating regularization terms into the loss function, thereby ensuring that all parameters in the model achieve practical identifiability. Additionally, we provide formal proof that the inclusion of this regularization guarantees the practical identifiability of all parameters (Details in Materials and Methods). Second, traditional methods for uncertainty quantification often involve perturbing all parameters simultaneously. This approach inadvertently modifies the loss function value, making it methodologically inconsistent, as uncertainty originates from non-identifiable parameters alone. To address this limitation, we develop an uncertainty quantification that focuses solely on non-identifiable parameters (Fig. 1). This method (Details in Materials and Methods) enables a more precise assessment of the influence of parameter uncertainty on model predictions.

The integration of mathematical models and data is essential in systems biology, yet determining how models can effectively guide data measurement remains a critical, unresolved challenge. For specific models, it is vital to design optimal experimental data collection strategies grounded in parameter practical identifiability^35^. Addressing this challenge, and leveraging our advancements in practical identifiability, we develop an algorithm to generate an optimal sequence of experimental measurement time points. This approach ensures that the collected data render all model parameters practically identifiable. We validate the algorithm by applying it to the Hill function (Fig. 3d) and the Michaelis–Menten system (Fig. 6b), successfully identifying critical data points that constitute the minimal dataset required for render parameter practical identifiability.

In conclusion, we present a novel framework for practical identifiability analysis, grounded in a rigorous new definition of practical identifiability. The framework systematically integrates the essential properties of practical identifiability and introduces innovative tools, such as novel regularization terms and uncertainty quantification methods. Building on these principles, we develop an algorithm designed to guide optimal data collection, ensuring that experimental data robustly supports model parameter practical identifiability. Our practical identifiability analysis framework demonstrates substantial potential as a crucial bridge between mathematical modeling and experimental data in systems biology.

## Materials and Methods

To support the subsequent mathematical description, we first introduce a novel definition of parameter practical identifiability and explain how it can be analyzed using the FIM. Second, we compare the FIM-based approach with an alternative method for assessing parameter coordinate identifiability via Bayesian posterior likelihood^36^. Third, to ensure all parameters are practically identifiable, we propose a novel regularization method using eigenvalue decomposition on the FIM and conduct uncertainty quantification for the non-identifiable parameters. Fourth, we discuss the relationship between structural identifiability and practical identifiability. Fifth, we propose a quantitative metric to evaluate the contribution of large datasets to parameter practical identifiability, and develop an algorithm to guide the optimal data collection for experiments, ensuring that all parameters in the model are practically identifiable with minimal data. Last, we perform practical identifiability analysis to two cases: function fitting, which includes polynomial fitting, Hill function, and neural network, and differential equations in diverse biological systems, including ODEs and PDEs.

### Practical Identifiability Analysis Using the FIM

For the time-series data-driven modeling approach, the loss function $l(h(\varphi (t,\theta )),\hat{h})$ is defined using the least squares method as follows:

(1) $$l\left(h\left(\varphi \left(t,\theta \right)\right),\hat{h}\right)=\overset{N}{\underset{i=1}{\sum }}{\left\|h\left(\varphi \left(t_{i},\theta \right)\right)-{\hat{h}}_{i}\right\|}_{2}^{2},$$

where N is the number of experimental data, $\varphi (t,\theta )\in R^{M}$ denotes the system output with parameter θ at the time $t={\left[t_{1},t_{2},\ldots ,t_{N}\right]}^{T}\;(\varphi (t,\theta )=\left.{\left[\varphi_{1}(t,\theta ),\varphi_{2}(t,\theta ),\ldots ,\varphi_{L}(t,\theta )\right]}^{\text{T}},\varphi_{i}(t)={\left[\varphi_{i}\left(t_{1},\theta \right),\varphi_{i}\left(t_{2},\theta \right),\ldots ,\varphi_{i}\left(t_{N},\theta \right)\right]}^{\text{T}}\right)$. The experimental observation is denoted as ${\left\{t_{i},{\hat{h}}_{i}\right\}}_{i=1}^{N}\left(\hat{h}={\left[{\hat{h}}_{1},{\hat{h}}_{2},\ldots ,{\hat{h}}_{N}\right]}^{\text{T}}\right)$, and the continuous differentiable function h(⋅) represents measurable quantities $\left(h(\cdot )\in R^{L}\right)$. The parameters of this system $\theta^{*}$ are given as

(2) $$\theta^{*}=\underset{\theta \in \Theta }{\text{argmin}}\;l(h(\varphi (t,\theta )),\hat{h}),$$

where Θ is the parameter space. The parameter of this system $\theta_{\delta }$ for the presence of small perturbation (δ) in measurements is obtained as

(3) $$\theta_{\delta }=\underset{\theta \in \Theta }{\text{argmin}}\;l(h(\varphi (t,\theta )),\hat{h}-\delta ).$$

Herein, the loss function $l(h(\varphi (t,\theta )),\hat{h}-\delta )$ is hypothesized to be continuous with respect to small perturbation (δ). We propose a novel definition of parameter practical identifiability based on the changes in parameters resulting from measurement perturbations (Eqs. (2)–(3)) as follows:

**Definition 1:** The parameter $\theta^{*}$ in Θ is practically identifiable if $\forall \varepsilon >0,\;\exists \Delta >0,\;\|\delta \|<\Delta ,\;\left\|\theta_{\delta }-\theta^{*}\right\|<\varepsilon$ where $\theta^{*}$ and $\theta_{\delta }$ satisfy Eq. (2) and Eq. (3), respectively.

Then we define the general sensitive matrix $s\left(\theta^{*}\right)$ with the observable function $h(\cdot )\in R^{L}$ as:

(4) $$s\left(\theta^{*}\right)={\left[s_{1}\left(\theta^{*}\right);s_{2}\left(\theta^{*}\right);\ldots ;s_{L}\left(\theta^{*}\right)\right]}_{L\times 1}\;s_{l}\left(\theta^{*}\right)={\left[\begin{array}{cccc} s_{l1}\left(t_{1}\right) & s_{l2}\left(t_{1}\right) & \cdots & s_{lk}\left(t_{1}\right)\\ s_{l1}\left(t_{2}\right) & s_{l2}\left(t_{2}\right) & \cdots & s_{lk}\left(t_{2}\right)\\ \vdots & \vdots & \ddots & \vdots \\ s_{l1}\left(t_{N}\right) & s_{l2}\left(t_{N}\right) & \cdots & s_{lk}\left(t_{N}\right) \end{array}\right]}_{N\times k},\;s_{li}\left(t_{j}\right)=\overset{M}{\underset{m=1}{\sum }}\frac{\partial h_{l}\left(\varphi \right)}{\partial \varphi_{m}}\frac{\partial \varphi_{m}\left(t_{j},\theta^{*}\right)}{\partial \theta_{i}}.$$

If the function h(⋅) is an identity mapping in M-dimensional space $\left(\frac{\partial h}{\partial \varphi }=I_{M},L=M\right)$, the general sensitivity matrix simplifies to the classical sensitivity matrix^29^ as follows:

$$s\left(\theta^{*}\right)={\left[s_{1}\left(\theta^{*}\right);s_{2}\left(\theta^{*}\right);\ldots ;s_{M}\left(\theta^{*}\right)\right]}_{M\times 1}\;s_{m}\left(\theta^{*}\right)={\left[\begin{array}{cccc} s_{m1}\left(t_{1}\right) & s_{m2}\left(t_{1}\right) & \cdots & s_{mk}\left(t_{1}\right)\\ s_{m1}\left(t_{2}\right) & s_{m2}\left(t_{2}\right) & \cdots & s_{mk}\left(t_{2}\right)\\ \vdots & \vdots & \ddots & \vdots \\ s_{m1}\left(t_{N}\right) & s_{m2}\left(t_{N}\right) & \cdots & s_{mk}\left(t_{N}\right) \end{array}\right]}_{N\times k},s_{mi}\left(t_{j}\right)=\frac{\partial \varphi_{m}\left(t_{j},\theta^{*}\right)}{\partial \theta_{i}}.$$

Then FIM is defined as follows:

(5) $$F\left(\theta^{*}\right)=\frac{1}{\sigma^{2}}s{\left(\theta^{*}\right)}^{T}s\left(\theta^{*}\right).$$

Based on **these definitions**, we explore the relationship between parameter practical identifiability and FIM as stated in **Theorem 1:**

**Theorem 1:** The parameter $\theta^{*}$ in Θ is practically identifiable if and only if the FIM $F\left(\theta^{*}\right)$ is invertible. (Details of the proof in the “Proof of Theorem 1“ section in Supplementary Materials)

### Coordinate Parameter Identifiability

Coordinate parameter identifiability is defined using the Bayesian posterior likelihood^28,36,37^ as follows:

**Definition 2:** The parameter θ is coordinate identifiable if the profile likelihood $PL\left(\hat{h}|\theta_{i}\right)=\underset{\theta_{j\neq i}}{\text{min}}[l(\hat{h};\theta )]$ has a locally unique minimum at $\theta_{i}^{*}$ for each parameter coordinate i..

Considering that the coordinate parameter identifiability is local, we use the linear approximation to investigate the relationship between the practical identifiability and coordinate identifiability at the given parameter point $\theta^{*}$. First, the observable quantities of the system h(φ(t,θ)) at the fixed time $t=t_{j}$ is linearly approached as:

(6) $$h\left(\varphi \left(t_{j},\theta \right)\right)\approx h\left(\varphi \left(t_{j},\theta^{*}\right)\right)+s_{j}\left(\theta^{*}\right)\left(\theta -\theta^{*}\right),$$

where $s_{j}\left(\theta^{*}\right)$ is defined in Eq. (4). The logarithmic likelihood function $l(\hat{h};\theta )\equiv -\text{log}ℒ(\hat{h};\theta )$ is given as

(7) $$\underset{\theta }{\text{min}\;}l\left(\hat{h};\theta \right)\approx \underset{\theta }{\text{min}}{\left\|h\left(\varphi \left(t,\theta^{*}\right)\right)+s\left(\theta^{*}\right)\left(\theta -\theta^{*}\right)-\hat{h}\right\|}_{2}^{2},$$

where $\;h\left(\varphi \left(t,\theta^{*}\right)\right)={\left[h\left(\varphi \left(t_{1},\theta \right)\right),h\left(\varphi \left(t_{2},\theta \right)\right),\ldots ,h\left(\varphi \left(t_{N},\theta \right)\right)\right]}^{T},h\left(\varphi \left(t_{i},\theta \right)\right)={\left[h_{1}\left(\varphi \left(t_{i},\theta \right)\right),h_{2}\left(\varphi \left(t_{i},\theta \right)\right),\ldots ,h_{L}\left(\varphi \left(t_{i},\theta \right)\right)\right]}^{T}$ is the observable system output with parameter θ and the experiment data is denoted as ${\left\{t_{i},{\hat{h}}_{i}\right\}}_{i=1}^{N}\;\left(\hat{h}={\left[{\hat{h}}_{1},{\hat{h}}_{2},\ldots ,{\hat{h}}_{N}\right]}^{\text{T}}\right)$. We denote the sensitive matrix at the parameter $\theta^{*}$ as $S=s\left(\theta^{*}\right)$ and the constant vector as $b=\hat{h}-h\left(\varphi \left(t,\theta^{*}\right)\right)+s\left(\theta^{*}\right)\theta^{*}$ so that Eq. (7) is rewritten as

(8) $$\underset{\theta }{\text{min}\;}l(\hat{h};\theta )\approx \underset{\theta }{\text{min}}\|S\theta -b{\|}_{2}^{2}.$$

**Theorem 2:** The parameter $\theta^{*}$ is coordinate identifiable if and only if the FIM $F\left(\theta^{*}\right)$ is invertible. (Details of the proof in the “Proof of Theorem 2“ section in Supplementary Materials)

If the FIM is singular, we further investigate the coordinate non-identifiability using the sensitive matrix $s\left(\theta^{*}\right)$ as follows:

**Theorem 3:** The parameter $\theta_{i}^{*}\in \theta^{*}$ is non-identifiable if and only if $s_{i}\in \text{range}(A)$. Here, $s_{i}$ is the $i^{\text{th}}$ column of matrix $s\left(\theta^{*}\right)$ and $A=\left[s_{1},s_{2},\cdots ,s_{i-1},s_{k},s_{i+1},\ldots ,s_{k-1}\right]$. In another word, A is the (k-1) column of matrix $\hat{S}=SP_{i,k}$, where $P_{i,k}=\left[e_{1},e_{2},\cdots ,e_{i-1},e_{k},e_{i+1},\ldots ,e_{k-1},e_{i}\right]$ is the elementary matrix and the vector $e_{i}$ is unite vector. (Details in ‘Proof of Theorem 3’ section in Supplementary Materials)

### Parameter Regularization and Uncertainty Quantification Based on Practical Identifiability

Based on Bayes’ theorem^51^, the likelihood function is extended by the prior probability density function (PDF) of the parameter P(θ) and the posterior PDF of the parameters is given as

(9) $$P(\theta |\hat{h})=\frac{P(\hat{h}|\theta )P(\theta )}{P(\hat{h})},P(\hat{h}|\theta )=ℒ(\hat{h};\theta )$$

where $P(\hat{h})$ is the PDF of the experimentally observable data. The parameter $\theta^{*}$ is obtained by maximum a posteriori (MAP) estimation as

(10) $$\theta^{*}=\underset{\theta \in \Theta }{\text{argmax}}\;\text{log}\;P(\theta |\hat{h})=\underset{\theta \in \Theta }{\text{argmin}}(-\text{log}ℒ(\hat{h};\theta )-\text{log}\;P(\theta )).$$

Herein, the prior PDF of the parameter P(θ) can be seen as the normalization to the parameter θ. The relative entropy is shown as follows:

(11) $$D\left(ℒ\left(\hat{h};\theta^{*}\right):ℒ\left(\hat{h}-\delta ;\theta_{\delta }\right)\right)=\frac{1}{2\sigma^{2}}\delta^{T}\delta +\frac{1}{\sigma^{2}}\delta^{T}\nabla h\left(\varphi \left(t,\theta^{*}\right)\right)-\frac{1}{2}{\left(\theta_{\delta }-\theta^{*}\right)}^{T}F_{\delta }\left(\theta^{*}\right)\left(\theta_{\delta }-\theta^{*}\right)$$

According to the limitation $\underset{\|\delta \|\to 0}{\text{lim}}D\left(ℒ(\hat{h};\theta ):ℒ\left(\hat{h}-\delta ;\theta_{\delta }\right)\right)=0$, we have $\left(\theta_{\delta }-\right.{\left.\theta^{*}\right)}^{T}F\left(\theta^{*}\right)\left(\theta_{\delta }-\theta^{*}\right)=0$. We perform the eigenvalue decomposition^52^ to FIM $F\left(\theta^{*}\right)$ as:

(12) $$F\left(\theta^{*}\right)=U\Sigma U^{T},\Sigma =\left[\begin{array}{cc} \Lambda_{r\times r} & 0\\ 0 & 0 \end{array}\right],U^{T}U=I_{k},U=\left[U_{r}U_{k-r}\right].$$

The Eq. (12) is transformed as:

(13) $${\left(\left[U_{r}^{T};U_{k-r}^{T}\right]\left(\theta_{\delta }-\theta^{*}\right)\right)}^{T}\left[\begin{array}{cc} \Lambda_{r\times r} & 0\\ 0 & 0 \end{array}\right]\left(\left[U_{r}^{T};U_{k-r}^{T}\right]\left(\theta_{\delta }-\theta^{*}\right)\right)=0$$

The Eq. (13) reflects that the (k-r) parameters are non-identifiable and that the r parameters are practical identifiable because of $\underset{\|\delta \|\to 0}{\text{lim}}\left\|U_{r}^{T}\left(\theta_{\delta }-\theta^{*}\right)\right\|=0$. Moreover, the prior PDF of the parameter P(θ) can be assumed as the gauss distribution at the low dimensional space $U_{k-r}^{T}\left(\theta -\theta^{*}\right)\sim N\left(0,\Sigma_{1}\right),\Sigma_{1}=\tau^{2}I_{k-r}$, and the gauss distribution of P(θ) is given as:

(14) $$P(\theta )=\frac{1}{(2\pi {)}^{\frac{k-r}{2}}{\left|U_{k-r}\Sigma_{1}U_{k-r}^{T}\right|}^{\frac{1}{2}}}\text{exp}\left(-\frac{1}{2}{\left(U_{k-r}^{T}\theta -U_{k-r}^{T}\theta^{*}\right)}^{T}{\left(U_{k-r}\Sigma_{1}U_{k-r}^{T}\right)}^{-1}\left(U_{k-r}^{T}\theta -U_{k-r}^{T}\theta^{*}\right)\right)$$

So, the regularization denoted as logP(θ) of the parameter $\theta_{i}^{*}$

(15) $$\text{log}\;P\left(\theta \right)=\text{log}\frac{1}{{\left(2\pi \tau^{2}\right)}^{\frac{k-r}{2}}}-\frac{1}{2\tau^{2}}{\left(U_{k-r}^{T}\theta -U_{k-r}^{T}\theta^{*}\right)}^{T}\left(U_{k-r}^{T}\theta -U_{k-r}^{T}\theta^{*}\right)\;=\text{log}\frac{1}{{\left(2\pi \tau^{2}\right)}^{\frac{k-r}{2}}}-\frac{1}{2\tau^{2}}{\left\|U_{k-r}^{T}\theta -U_{k-r}^{T}\theta^{*}\right\|}_{2}^{2}$$

For the parameter θ, the regularization without constant part is given as $\lambda {\left\|U_{k-r}^{T}\theta -U_{k-r}^{T}\theta^{*}\right\|}_{2}^{2}\left(\lambda =\frac{1}{2\tau^{2}}\right)$. The MAP estimation is rewritten as

(16) $$\tilde{\theta }=\underset{\theta \in \Theta }{\text{argmin}}\left(-\text{log}\;ℒ(\hat{h};\theta )+\lambda {\left\|U_{k-r}^{T}\theta -U_{k-r}^{T}\theta^{*}\right\|}_{2}^{2}\right)$$

$\tilde{\theta }$ is practically identifiable because the necessary condition of Eq. (16) is $\left(\lambda U_{k-r}U_{k-r}^{T}+S^{T}S\right)\tilde{\theta }=S^{T}b$. The FIM of the parameter $\tilde{\theta }$ is

(17) $$F\left(\tilde{\theta }\right)=\lambda U_{k-r}U_{k-r}^{T}+S^{T}S=U\left[\begin{array}{cc} \Lambda_{r\times r} & 0\\ 0 & \lambda I_{k-r} \end{array}\right]U^{T}.$$

$F(\tilde{\theta })$ is full rank, and the parameter $\tilde{\theta }$ is coordinate identifiable according to **Theorem 2**.

We propose an uncertainty quantification method based on practical identifiability to examine the impact of variations in the non-identifiable parameters on the model’s uncertainty, ensuring that the observations remain within the defined confidence intervals. To address uncertainties in the parameters, especially those aligned with the non-identifiable eigenvectors $U_{k-r}^{T}$, we perform a perturbation vector as $\varepsilon_{k-r}\sim N\left(0,\Sigma_{k-r}\right)\left(U_{k-r}^{T}\hat{\theta }=U_{k-r}^{T}\tilde{\theta }+\varepsilon_{k-r}\right)$. The model parameters are adjusted by:

(18) $$\hat{\theta }=\tilde{\theta }+U_{k-r}\varepsilon_{k-r}.$$

The observable variable $h(\varphi (t,\bar{\theta }))$ is linearly approached as

(19) $$h(\varphi (t,\hat{\theta }))\approx h(\varphi (t,\tilde{\theta }))+\nabla_{\theta }h(\varphi (t,\tilde{\theta }))(\hat{\theta }-\tilde{\theta }).$$

Based on law of propagation of uncertainty^53^, the estimation of uncertainty on the observable variable $h_{l}(\varphi (t,\hat{\theta }))(l=1,2,\ldots ,L)$ is written as:

(20) $$\text{Var}\left(h_{l}(\varphi (t,\hat{\theta }))\right)=\nabla_{\theta }h_{l}(\varphi (t,\tilde{\theta }))CoV(\hat{\theta }){\left(\nabla_{\theta }h_{l}\left(\varphi \left(t,\tilde{\theta }\right)\right)\right)}^{T},\forall t>0.$$

where the variance of parameter $\bar{\theta }$ is obtain as

$$CoV\left(\hat{\theta }\right)=U_{k-r}\Sigma_{k-r}U_{k-r}^{T}.$$

Through the linear approximation, the variance of the state variable is calculated using the error propagation formula, which can then be used to construct the confidence interval for the state variable. Assuming each component of observable variable $h_{l}(\varphi (t,\hat{\theta }))(l=1,2,\ldots ,L)$ approximately follows a normal distribution, its 100(1-α)% confidence interval follows:

(21) $$h_{l}(\varphi (t,\hat{\theta }))\in \left[h_{l}(\varphi (t,\tilde{\theta }))-z_{\frac{\alpha }{2}}\sqrt{Var\left(h_{l}(\varphi (t,\hat{\theta }))\right)},(h_{l}\varphi (t,\tilde{\theta })+z_{\frac{\alpha }{2}}\sqrt{Var\left(h_{l}(\varphi (t,\hat{\theta }))\right)}\right],$$

where $Z_{\frac{\alpha }{2}}$ is the critical value of the standard normal distribution.

### Structural Identifiability vs. Practical Identifiability

The definition of structural identifiability is stated as follows^29^:

**Definition 3:** The parameter $\theta^{*}$ in Θ is structural identifiability if $\exists \delta >0,\;\forall \theta \in U\left(\theta^{*},\delta \right)$, the following property holds

(22) $$\forall t>0,h(\varphi (t,\theta ))=h\left(\varphi \left(t,\theta^{*}\right)\right)\Rightarrow \theta =\theta^{*}$$

**Theorem 4:** The parameter $\theta^{*}$ in Θ is structural identifiable if and only if $\forall {\left\{t_{i}\right\}}_{i=1}^{\infty }$, there is a subsequence ${\left\{t_{i_{j}}\right\}}_{j=1}^{M}(M=L*N\geq k)$, and $s\left(\theta^{*}\right)$ has column full rank. Herein, $s\left(\theta^{*}\right)$ is the Jacobian matrix to the parameter $\theta^{*}$ at the sequence ${\left\{t_{i_{j}}\right\}}_{j=1}^{M}$ and k is the number of parameters.

### Quantifying Dataset Contributions to Parameters and Optimizing Data Collection

#### Quantification of dataset contribution to parameter practical identifiability.

We propose a quantitative metric (ξ) to evaluate the contribution of a dataset to the practical identifiability of model parameters. The index ξ is defined as the ratio of the smallest eigenvalue $\left(\sigma_{\text{min}}\right)$ to the largest eigenvalue of the FIM $\left(\sigma_{\text{max}}\right)$ as follows:

(23) $$\xi =\frac{\sigma_{\text{min}}}{\sigma_{\text{max}}}$$

As the dataset size increases, ξ approaches a steady state, which represents the maximum contribution of the dataset to the practical identifiability of the model parameters.

#### Optimal Data Collection Design.

We develop an optimization algorithm to ensure that all parameters in the model are practically identifiable using the minimal amount of data. The algorithm aims to find a minimal time series ${\left\{t_{j}\right\}}_{j=1}^{m}$ at which the minimum eigenvalue of matrix $F\left(\theta^{*}\right)$ exceed eigenvalue tolerance ε. We find a time point $t_{j}$ and a sequence ${\left\{\alpha_{i}\right\}}_{i=1}^{k}$ satisfying the following equation:

$$\nabla_{\theta }h_{l}\left(\varphi \left(t_{j},\theta^{*}\right)\right)=\sum_{i=1}^{k}\alpha_{i}U_{i}\left(\alpha_{j}\neq 0,i=r+1,\ldots ,k,l=1,2,\ldots ,L\right),$$

where ${\left\{U_{i}\right\}}_{i=1}^{k}$ is the column vector of matrix U obtained by the eigenvalue decomposition on the FIM $F\left(\theta^{*}\right)$ at the time-series points ${\left\{t_{j}\right\}}_{j=1}^{m}$ as:

(24) $$F\left(\theta^{*}\right)=s^{T}\left(\theta^{*}\right)s\left(\theta^{*}\right)=U_{k\times k}\Sigma_{k\times k}U_{k\times k}^{T},\;s\left(\theta^{*}\right)={\left[\begin{array}{c} \tilde{h}\left(\varphi \left(t_{1},\theta^{*}\right)\right)\\ \tilde{h}\left(\varphi \left(t_{2},\theta^{*}\right)\right)\\ \vdots \\ \tilde{h}\left(\varphi \left(t_{m},\theta^{*}\right)\right) \end{array}\right]}_{(m*L)\times k}$$

$$\Sigma_{k\times k}=\text{diag}(s_{1},s_{2},\ldots ,s_{r},\underset{k-r}{\underbrace{0,\ldots ,0}})\left(s_{r}\neq 0,r\leq k\right)$$

$$\begin{array}{cc} & \tilde{h}\left(\varphi \left(t_{j},\theta^{*}\right)\right)={\left[\begin{array}{c} \nabla_{\theta }h_{1}{\left(\varphi \left(t_{j},\theta^{*}\right)\right)}^{T}\\ \nabla_{\theta }h_{2}{\left(\varphi \left(t_{j},\theta^{*}\right)\right)}^{T}\\ \vdots \\ \nabla_{\theta }h_{L}{\left(\varphi \left(t_{j},\theta^{*}\right)\right)}^{T} \end{array}\right]}_{L\times k},j=1,2,\ldots ,m \end{array}$$

The question on finding the time point t and the sequence ${\left\{\alpha_{i}\right\}}_{i=1}^{k}$ satisfying the formula $\nabla_{\theta }h_{l}\left(\varphi \left(t_{m},\theta^{*}\right)\right)=\sum_{i=1}^{k}\alpha_{il}U_{i}\left(\alpha_{jl}\neq 0,j=r+1,\ldots ,k,l=1,2,\ldots ,L\right)$ is transformed as an optimization as:

(25) $$\underset{t}{\text{min}}\overset{k}{\underset{j=r+1}{\sum }}\overset{L}{\underset{l=1}{\sum }}{\left\|\frac{\nabla_{\theta }h_{l}\left(\varphi \left(t,\theta^{*}\right)\right)-\sum_{i=1}^{k}\alpha_{il}U_{i}}{\alpha_{jl}}\right\|}_{2}^{2},\alpha_{il}=\langle \nabla_{\theta }h_{l}{\left(\varphi \left(t,\theta^{*}\right)\right)}^{T},U_{i}\rangle .$$

The algorithm is presented as follows:

**Algorithm 1:** Practical Identifiability of small datasets via Eq. (25)

**Input:** Model φ(t,θ), observation h(⋅), tolerance ε
**Output:** Time set T
1. Choose randomly q time points as an initialized time series $T={\left\{t_{j}\right\}}_{j=1}^{q}$, denote the size of time series m=q, eigenvalue tolerance ε, and maximum iteration number as M;
2. Perform the eigenvalue decomposition on the Jacobian matrix $F\left(\theta^{*}\right)$ as: $F\left(\theta^{*}\right)=U\Sigma U^{T}$
**3**. While the total step reaches the maximum iteration (m-k≥M) or $\Sigma_{kk}\geq \varepsilon$ **DO**
4. Find t∉T through the optimization
$$\underset{t}{min}\overset{k}{\underset{j=r+1}{\sum }}\overset{L}{\underset{l=1}{\sum }}{\left\|\frac{\nabla_{\theta }h_{l}\left(\varphi \left(t,\theta^{*}\right)\right)-\sum_{i=1}^{k}\alpha_{il}U_{i}}{\alpha_{jl}}\right\|}_{2}^{2}\;\alpha_{il}=\langle \nabla_{\theta }h_{l}{\left(\varphi \left(t,\theta^{*}\right)\right)}^{T},U_{i}\rangle ,i=1,2,\ldots ,k,l=1,2,\ldots ,L\;m=m+1$$
5. Put the time t into the set T as $t_{m}$ and m is the size of time series ${\left\{t_{j}\right\}}_{j=1}^{m}$. Perform the eigenvalue decomposition on the Jacobian matrix $F\left(\theta^{*}\right)$ as $F\left(\theta^{*}\right)=s{\left(\theta^{*}\right)}^{T}s\left(\theta^{*}\right)$
**6. END WHILE**
**7. Return:** Time set $T={\left\{t_{j}\right\}}_{j=1}^{m}$

### Applications of Practical Identifiability

We construct three computing examples, such as polynomial fitting, Hill function fitting, and neural network fitting, to illustrate the superiority of FIM to analyze the practical identifiability.

#### Polynomial fitting example.

Polynomial fitting example is constructed as follows

(26) $$\varphi (t,\theta )=\theta_{1}+\theta_{2}t^{2}+\theta_{3}[(t-1)(t-2)(t-3)+2],h(\varphi )=\varphi .$$

And the synthetic data is as follows:

**Table:** 

$t_{j}$	1	2	3
${\overset{ˆ}{h}}_{j}$	4	7	12

#### Hill function example.

The formular of Hill function follows:

(27) $$\varphi \left(x,\theta \right)=V_{max}\frac{x^{n}}{x^{n}+K_{d}^{n}},\theta =\left[V_{max},K_{d},n\right],h\left(\varphi \right)=\varphi .$$

“The synthetic dataset is presented as follows:

**Table:** 

$x_{j}$	1.6	3.2	4.8	6.4	8.0
${\overset{ˆ}{h}}_{j}$	0.0065	0.6263	0.9772	0.9977	0.9996

#### Neural network example.

A neural network is constructed with one hidden layer denoted as φ(t,θ) to fit the function $\overset{ˆ}{h}(t)=\text{sin}(2\pi t)(t\in [0,1])$. The synthetic data is generated by uniformly sampling N points over the interval [0,1] denoted as ${\left\{t_{i},\overset{ˆ}{h}\left(t_{i}\right)\right\}}_{i=1}^{N}$. The logarithmic likelihood function is the least square formular as follows:

$$l(y;\theta )=\frac{1}{N}\overset{N}{\underset{i=1}{\sum }}{\left(\varphi \left(t_{i},\theta \right)-\text{sin}\left(2\pi t_{i}\right)\right)}^{2},t_{i}=\frac{i-1}{N-1};$$

(28) $$\varphi (t,\theta )=\overset{M}{\underset{j=1}{\sum }}w_{j}\sigma \left(\alpha_{j}t+\beta_{j}\right).$$

The parameter is $\theta =\left[\alpha_{1},\alpha_{2},\ldots ,\alpha_{M},\beta_{1},\beta_{2},\ldots ,\beta_{M},w_{1},w_{2},\ldots ,w_{M}\right]$, and the sensitive matrix is conducted as follows:

$$\nabla \varphi \left(t_{i},\theta \right)=\left[\begin{array}{lll} \nabla \varphi \left(t_{i},\alpha \right) & \nabla \varphi \left(t_{i},\beta \right) & \nabla \varphi \left(t_{i},\omega \right) \end{array}\right]$$

$$\nabla \varphi \left(t_{i},\alpha \right)=\left[w_{1}\frac{\partial \sigma \left(\alpha_{1}t_{i}+\beta_{1}\right)}{\partial \alpha_{1}},w_{2}\frac{\partial \sigma \left(\alpha_{2}t_{i}+\beta_{2}\right)}{\partial \alpha_{2}},\ldots ,w_{M}\frac{\partial \sigma \left(\alpha_{M}t_{i}+\beta_{M}\right)}{\partial \alpha_{M}}\right],$$

$$\nabla \varphi \left(t_{i},\beta \right)=\left[w_{1}\frac{\partial \sigma \left(\alpha_{1}t_{i}+\beta_{1}\right)}{\partial \beta_{1}},w_{2}\frac{\partial \sigma \left(\alpha_{2}t_{i}+\beta_{2}\right)}{\partial \beta_{2}},\ldots ,w_{M}\frac{\partial \sigma \left(\alpha_{M}t_{i}+\beta_{M}\right)}{\partial \beta_{M}}\right],$$

$$\nabla \varphi \left(t_{i},\omega \right)=\left[\sigma \left(\alpha_{1}t_{i}+\beta_{1}\right),\sigma \left(\alpha_{2}t_{i}+\beta_{2}\right),\ldots ,\sigma \left(\alpha_{M}t_{i}+\beta_{M}\right)\right].$$

We leverage two different activation functions, namely ReLU and tanh as follows:

case (1) $$\text{ReLu}\left(x\right)=\left\{\begin{array}{ll} x, & x\geq 0\\ 0, & x<0 \end{array},\frac{\text{dReLU}\left(x\right)}{dx}=\left\{\begin{array}{ll} 1, & x\geq 0\\ 0, & x<0 \end{array}\right.\right.;$$

case (2) $$\frac{d\text{tanh}(x)}{dx}=1-{\text{tanh}}^{2}(x)$$

In case (1), N is chosen as 500, and M is set to 40. In case (2), N is decreased to 20 because of the smoothness of tanh.

#### Various biological systems.

Practical identifiability analysis is widely applied to dynamic differential equation models in biological systems to assess whether model parameters can be reliably identified from available data. In this study, we evaluate the proposed parameter practical identifiability metric by leveraging it to five dynamic differential equation models: the LV model^41^, the Michaelis-Menten system^43^, the SEIR model^45^, a cascade model of Alzheimer’s disease (AD)^46^, and PDE model of cancer-immune interaction model^47^. A general form of ODEs is described as:

$$\frac{d\varphi }{dt}=f\left(\varphi ,\theta \right),$$

(29) h(t,θ)=h(φ(t,θ)).

Herein, $\varphi (t,\theta )\in R^{m}$ is a vector of state variables, $h(t,\theta )\in R^{L}$ is the measurement or output vector. $\theta \in R^{k}$ is parameter vector and assumed as constants in this work. Let $\varsigma (t,\theta )=\frac{\partial \varphi }{\partial \theta }$, it can be shown that ς satisfies

(30) $$\frac{d\varsigma }{dt}=\frac{\partial f(\varphi ,\theta )}{\partial \theta }+J(\varphi ,\theta )\varsigma ,$$

$$\varsigma \left(0,\theta \right)=0.$$

Where J(φ,θ) is the Jacobian $\frac{\partial f(\varphi ,\theta )}{\partial \varphi }$. The measurement h(t,θ) satisfies $\frac{d}{dt}\frac{\partial h}{\partial \theta }=\frac{\partial h}{\partial \varphi }\frac{d\varsigma }{dt}$ so that the sensitive matrix s(θ) is obtained by Eqs. (29–30) as follows:

$$\frac{d\varphi }{dt}=f\left(\varphi ,\theta \right),$$

$$\frac{d\varsigma }{dt}=\frac{\partial f(\varphi ,\theta )}{\partial \theta }+J\left(\varphi ,\theta \right)\varsigma ,$$

(31) $$\frac{d}{dt}\frac{\partial h}{\partial \theta }=\frac{\partial h}{\partial \varphi }\frac{d\varsigma }{dt},$$

$$h\left(t,\theta \right)=h\left(\varphi \left(t,\theta \right)\right),$$

$$\varsigma \left(0,\theta \right)=0,\varphi \left(0,\theta \right)=\varphi_{0}.$$

#### LV model.

The classical LV model^41^ describes the dynamics of prey and predator as follows:

$$\frac{dx}{dt}=\alpha x-\beta xy,$$

(32) $$\frac{dy}{dt}=\delta xy-\gamma y.$$

Herein, the parameter is $\theta =[\alpha ,\beta ,\delta ,\gamma {]}^{T}$ and the observable variable is h(t,θ)=[x(t,θ),y(t,θ)]. The variable x and y represent the prey and predator, respectively.

#### Michaelis–Menten system.

The Michaelis-Menten system^44^ is used to model the enzyme reaction or ligand-receptor response as follows:

$$\frac{d[S]}{dt}=-k_{1}\left[S\right]\left[E\right]+k_{2}\left[ES\right],$$

$$\frac{d[E]}{dt}=-k_{1}\left[S\right]\left[E\right]+\left(k_{2}+k_{3}\right)\left[ES\right],$$

$$\frac{d[ES]}{dt}=k_{1}\left[S\right]\left[E\right]-\left(k_{2}+k_{3}\right)\left[ES\right],$$

(33) $$\frac{d[P]}{dt}=k_{3}\left[ES\right].$$

Herein, $\theta ={\left[k_{1},k_{2},k_{3}\right]}^{T}$. We consider two cases of observable variable as follows:

**case (1)** $\;h(t,\theta )=\left[y_{1}(t,\theta ),y_{4}(t,\theta )\right];$
**case (2)** $\;h\left(t,\theta \right)=y_{4}\left(t,\theta \right).$

#### SEIR model.

The SEIR model is the classical compartmental model to understand the disease dynamics^45^ as follows:

$$\frac{dS}{dt}=-\beta SI,$$

$$\frac{dE}{dt}=\beta SI-\sigma E,$$

$$\frac{dI}{dt}=\sigma E-\gamma I,$$

(34) $$\frac{dR}{dt}=\gamma I.$$

Herein $\theta =[\beta ,\sigma ,\gamma {]}^{T}$. There are four cases of observable as follows:

**case (1)** $\;h\left(t,\theta \right)=I\left(t,\theta \right);$
**case (2)** $\;h\left(t,\theta \right)=\left[E\left(t,\theta \right),I\left(t,\theta \right)\right];$
**case (3)** $\;h\left(t,\theta \right)=\left[S\left(t,\theta \right),I\left(t,\theta \right)\right];$
**case (4)** $\;h\left(t,\theta \right)=\left[S\left(t,\theta \right),E\left(t,\theta \right),I\left(t,\theta \right)\right].$

#### Cascade model of Alzheimer’s Disease.

Our group previously developed the cascade model of Alzheimer’s Disease^46^ as follows:

$$\frac{dA_{\beta }}{dt}=\lambda_{A_{\beta }}A_{\beta }\left(1-\frac{A_{\beta }}{K_{A_{\beta }}}\right),$$

$$\frac{d\tau_{p}}{dt}=\lambda_{\tau }A_{\beta }\left(1-\frac{\tau_{p}}{K_{\tau_{p}}}\right),$$

$$\frac{dN}{dt}=\lambda_{N_{\tau_{p}}}\tau_{p}\left(1-\frac{N}{K_{N}}\right),$$

(35) $$\frac{dC}{dt}=\left(\lambda_{CN}N+\lambda_{C_{\tau }}\tau_{p}\right)\left(1-\frac{C}{K_{C}}\right).$$

Where the observable variable is $h(t,\theta )=\left[A_{\beta }(t,\theta ),\tau_{p}(t,\theta ),N(t,\theta ),C(t,\theta )\right]$, and the parameter is $\theta ={\left[\lambda_{A_{\beta }},\lambda_{\tau },\lambda_{N_{\tau_{p}}},\lambda_{CN},\lambda_{C_{\tau }},K_{A_{\beta }},K_{\tau_{p}},K_{N},K_{C}\right]}^{T}$.

#### PDE model of cancer-immune interactions:

The model of tumor-immune interactions consists of three dependent variables denoted E,T and C, which are the local densities/concentrations of tumor-infiltrating cytotoxic lymphocytes (TICLs), tumor cells, TICL–tumor cell complexes, respectively. The formula of nondimensional model^47^ follows as:

$$\frac{\partial E}{\partial \overset{‾}{t}}=\nabla^{2}E+\sigma \chi \left(x\right)+\frac{\rho C}{\eta +T}-\sigma E-\mu ET+\epsilon C,$$

$$\frac{\partial T}{\partial \overset{‾}{t}}=\omega \nabla^{2}T+\beta_{1}T\left(1-\beta_{2}T\right)-\phi ET+\lambda C,$$

(36) $$\frac{\partial C}{\partial \overset{‾}{t}}=\mu ET-\psi C.$$

$$\chi (x)=\left\{\begin{array}{ll} 0, & \text{if}\;x\leq l=0.2\\ 1, & \text{if}\;x>l=0.2 \end{array},x\in [0,1]\right.$$

The initial conditions are given by

$$E(x)=\left\{\begin{array}{c} 0,\;x\leq l\\ \left(1-\text{exp}\left(-1000(x-l{)}^{2}\right)\right),x>l \end{array}\right.$$

$$T(x)=\left\{\begin{array}{c} T_{0}\left(1-\text{exp}\left(-1000(x-l{)}^{2}\right)\right),x\leq l\\ 0,\;x>l \end{array}\right.$$

$$C(x)=C_{0}\;\text{exp}\left(-1000(x-l{)}^{2}\right)$$

We use the non-flux boundary conditions as follows:

$$\frac{\partial E}{\partial x}(0,t)=\frac{\partial E}{\partial x}(1,t)=\frac{\partial T}{\partial x}(0,t)=\frac{\partial T}{\partial x}(1,t)=\frac{\partial C}{\partial x}(0,t)=\frac{\partial C}{\partial x}(1,t)=0$$

The parameter is $\theta ={\left[\sigma ,\rho ,\eta ,\mu ,\epsilon ,\beta_{1},\beta_{2},\phi ,\lambda ,\mu ,\psi \right]}^{T}$ and there are four observable variables we consider as follows:

**case (1)** $h\left(t,\theta \right)=\left[\int_{0}^{1}E\left(t,x;\theta \right)dx,\int_{0}^{1}T\left(t,x;\theta \right)dx\right]$
**case (2)** $\;h\left(t,\theta \right)=E\left(t,x;\theta \right),$
$$E(t,x;\theta )=\left[E\left(t,x_{0};\theta \right),E\left(t,x_{2};\theta \right),\ldots ,E\left(t,x_{N};\theta \right)\right],x_{i}=\frac{i}{N},i=0,1,2,\ldots ,N$$
**case (3)** $\;h\left(t,\theta \right)=T\left(t,x;\theta \right),$
$$T(t,x;\theta )=\left[T\left(t,x_{0};\theta \right),T\left(t,x_{2};\theta \right),\ldots ,T\left(t,x_{N};\theta \right)\right],x_{i}=\frac{i}{N},i=0,1,2,\ldots ,N$$
**case (4)** $\;h\left(t,\theta \right)=\left[E\left(t,x;\theta \right),T\left(t,x;\theta \right)\right],$
$$E\left(t,x;\theta \right)=\left[E\left(t,x_{0};\theta \right),E\left(t,x_{2};\theta \right),\ldots ,E\left(t,x_{N};\theta \right)\right],$$
$$T(t,x;\theta )=\left[T\left(t,x_{0};\theta \right),T\left(t,x_{2};\theta \right),\ldots ,T\left(t,x_{N};\theta \right)\right],x_{i}=\frac{i}{N},i=0,1,2,\ldots ,N$$

## Supplementary Material

1

## Data Availability

All relevant data are within the manuscript and its Supporting Information files. The public datasets are used in this study. Source codes and data have been deposited on the GitHub repository (https://github.com/WilliamMoriaty/Practical-Identifiability).

## List of abbreviation

FIM: Fisher information Matrix
EVD: Eigenvalue decomposition
AD: Alzheimer’s disease
PDF: Probability density function
ODE: Ordinary differential equation
PDE: Partial differential equation
LV: Lotka-Volterra
