# Supplementary material for: A Systematic Computational Framework for Practical Identifiability Analysis in Mathematical Models arising from biology
Source: ArXiv. 2025 Jan 2:arXiv:2501.01283v1. Preprint. [Version 1] (PMC11722522)
Supplement: Supplementary file 1 [file NIHPP2501.01283v1-supplement-1.pdf]

A Novel and Systematic Framework for Practical Identifiability Analysis in Systems Biological Mathematical Models

Shun Wang[1], Wenrui Hao[1,*]

Content

Section 1 Theoretical Analysis 1

Proof of Theorem 1 1

Proof of Theorem 2 4

Proof of Theorem 3 5

Proof of Theorem 4 6

Section 2 Supplementary Figures 6

Section 3 Values of the parameters 9

Section 4 Supplementary Tables 11

References 12

Section 1 Theoretical Analysis

Proof of Theorem 1

Proof: Before starting the proof, we set $L = 1$ for the convenience of the subsequent proof. The elements of the FIM are defined as

$$F(\boldsymbol{\theta}) = \{F(\boldsymbol{\theta})_{ij}\}_{i,j=1,2,\dots,k} = E\left(\frac{\partial \log \mathcal{L}(\widehat{\boldsymbol{h}}; \boldsymbol{\theta})}{\partial \theta_i} \frac{\partial \log \mathcal{L}(\widehat{\boldsymbol{h}}; \boldsymbol{\theta})}{\partial \theta_j}\right), \qquad (S1)$$

Where $\mathcal{L}(\widehat{\boldsymbol{h}}; \boldsymbol{\theta})$ is the likelihood function $\left(\mathcal{L}(\widehat{\boldsymbol{h}}; \boldsymbol{\theta}) > 0, \int \mathcal{L}(\widehat{\boldsymbol{h}}; \boldsymbol{\theta}) d\widehat{\boldsymbol{h}} = 1\right)$. We assume that $F(\boldsymbol{\theta})$ exist and are continuous functions of $\boldsymbol{\theta}$ in the parameter space Θ (*1*).

Assuming that the experiment measurements are contaminated by additive normally distributed uncorrelated random measurement errors (*2*), i.e. $\hat{h}_i =$

$h(\boldsymbol{\varphi}(t_i,\boldsymbol{\theta})) + \varepsilon_i, \varepsilon_i \sim \mathcal{N}(0,\sigma^2)$, the estimation of the model parameters is formulated as the maximization of the likelihood of the data as follows:

$$\mathcal{L}(\widehat{\boldsymbol{h}};\boldsymbol{\theta}) = \frac{1}{((2\pi)^N|\Sigma|)^{1/2}}\exp\left(-\frac{1}{2}\left(\widehat{\boldsymbol{h}} - \boldsymbol{h}(\boldsymbol{\varphi}(\boldsymbol{t},\boldsymbol{\theta}))\right)^T \Sigma^{-1}\left(\widehat{\boldsymbol{h}} - \boldsymbol{h}(\boldsymbol{\varphi}(\boldsymbol{t},\boldsymbol{\theta}))\right)\right), (S2)$$

where the vector $\boldsymbol{h}(\boldsymbol{\varphi}(\boldsymbol{t},\boldsymbol{\theta})) = \left(h(\boldsymbol{\varphi}(t_1,\boldsymbol{\theta})), h(\boldsymbol{\varphi}(t_2,\boldsymbol{\theta})), \dots, h(\boldsymbol{\varphi}(t_N,\boldsymbol{\theta}))\right)^T$ is the system output with parameter $\boldsymbol{\theta}$ and the experiment data is denoted as $\widehat{\boldsymbol{h}} = (\hat{h}_1, \hat{h}_2, \dots, \hat{h}_N)^T$. The covariance matrix $\Sigma = \sigma^2 I$, and I is the identity matrix. The logarithmic likelihood function $l(\widehat{\boldsymbol{h}};\boldsymbol{\theta}) \equiv -\log\mathcal{L}(\widehat{\boldsymbol{h}};\boldsymbol{\theta})$ is given as

$$l(\widehat{\boldsymbol{h}};\boldsymbol{\theta}) = \frac{1}{2\sigma^2}\left(\widehat{\boldsymbol{h}} - \boldsymbol{h}(\boldsymbol{\varphi}(\boldsymbol{t},\boldsymbol{\theta}))\right)^T\left(\widehat{\boldsymbol{h}} - \boldsymbol{h}(\boldsymbol{\varphi}(\boldsymbol{t},\boldsymbol{\theta}))\right) + \frac{N}{2}\log 2\pi + \frac{1}{2}\log\sigma^2 . (S3)$$

For the small perturbation $(\boldsymbol{\delta})$ in experimental data, the logarithmic likelihood function $l(\widehat{\boldsymbol{h}} - \boldsymbol{\delta};\boldsymbol{\theta})$ is changed as:

$$l(\widehat{\boldsymbol{h}} - \boldsymbol{\delta};\boldsymbol{\theta}) = \frac{1}{2\sigma^2}\left(\widehat{\boldsymbol{h}} - \boldsymbol{h}(\boldsymbol{\varphi}(\boldsymbol{t},\boldsymbol{\theta}))\right)^T\left(\widehat{\boldsymbol{h}} - \boldsymbol{h}(\boldsymbol{\varphi}(\boldsymbol{t},\boldsymbol{\theta}))\right)$$

$$-\frac{1}{\sigma^2}\boldsymbol{\delta}^T\left(\widehat{\boldsymbol{h}} - \boldsymbol{h}(\boldsymbol{\varphi}(\boldsymbol{t},\boldsymbol{\theta}))\right) + \frac{1}{2\sigma^2}\boldsymbol{\delta}^T\boldsymbol{\delta} + \frac{N}{2}\log 2\pi + \frac{1}{2}\log\sigma^2. \ (S4)$$

The relative entropy is utilized to measure the distribution distance between $\mathcal{L}(\widehat{\boldsymbol{h}};\boldsymbol{\theta}^*)$ and $\mathcal{L}(\widehat{\boldsymbol{h}} - \boldsymbol{\delta};\boldsymbol{\theta}_{\boldsymbol{\delta}})$ as

$$D\left(\mathcal{L}(\widehat{\boldsymbol{h}};\boldsymbol{\theta}^*):\mathcal{L}(\widehat{\boldsymbol{h}} - \boldsymbol{\delta};\boldsymbol{\theta}_{\boldsymbol{\delta}})\right) = \int \mathcal{L}(\widehat{\boldsymbol{h}};\boldsymbol{\theta}^*)\log\frac{\mathcal{L}(\widehat{\boldsymbol{h}};\boldsymbol{\theta}^*)}{\mathcal{L}(\widehat{\boldsymbol{h}} - \boldsymbol{\delta};\boldsymbol{\theta}_{\boldsymbol{\delta}})}d\widehat{\boldsymbol{h}}, (S5)$$

Using the Taylor expansion to second order, we have

$$D\left(\mathcal{L}(\widehat{\boldsymbol{h}};\boldsymbol{\theta}^*):\mathcal{L}(\widehat{\boldsymbol{h}} - \boldsymbol{\delta};\boldsymbol{\theta}_{\boldsymbol{\delta}})\right) = \int \mathcal{L}(\widehat{\boldsymbol{h}};\boldsymbol{\theta}^*)\,(\log\mathcal{L}(\widehat{\boldsymbol{h}};\boldsymbol{\theta}^*)$$

$$-\log\mathcal{L}(\widehat{\boldsymbol{h}} - \boldsymbol{\delta};\boldsymbol{\theta}^*) - \nabla_{\boldsymbol{\theta}}\log\mathcal{L}(\widehat{\boldsymbol{h}} - \boldsymbol{\delta};\boldsymbol{\theta}^*)^T\,(\boldsymbol{\theta}_{\boldsymbol{\delta}} - \boldsymbol{\theta}^*)$$

$$-\frac{1}{2}(\boldsymbol{\theta}_{\boldsymbol{\delta}} - \boldsymbol{\theta}^*)^T H\left(\log\mathcal{L}(\widehat{\boldsymbol{h}} - \boldsymbol{\delta};\boldsymbol{\theta}^*)\right)(\boldsymbol{\theta}_{\boldsymbol{\delta}} - \boldsymbol{\theta}^*) + o(\|\boldsymbol{\theta}_{\boldsymbol{\delta}} - \boldsymbol{\theta}^*\|^2)\boldsymbol{d}\widehat{\boldsymbol{h}}. (S6)$$

where $H\left(\log\mathcal{L}(\widehat{\boldsymbol{h}} - \boldsymbol{\delta};\boldsymbol{\theta}^*)\right)$ is the Hessian matrix of $l(\widehat{\boldsymbol{h}} - \boldsymbol{\delta};\boldsymbol{\theta}^*)$. Hence, these formulars can be given as

$$\int \mathcal{L}(\widehat{\boldsymbol{h}};\boldsymbol{\theta}^*)\left(\log\mathcal{L}(\widehat{\boldsymbol{h}};\boldsymbol{\theta}^*) - \log\mathcal{L}(\widehat{\boldsymbol{h}} - \boldsymbol{\delta};\boldsymbol{\theta}^*)\right)\boldsymbol{d}\widehat{\boldsymbol{h}}$$

$$= E\left[-\frac{1}{\sigma^2}\boldsymbol{\delta}^T\left(\widehat{\boldsymbol{h}} - \boldsymbol{h}(\boldsymbol{\varphi}(\boldsymbol{t},\boldsymbol{\theta}^*))\right) + \frac{1}{2\sigma^2}\boldsymbol{\delta}^T\boldsymbol{\delta}\right] = \frac{1}{2\sigma^2}\boldsymbol{\delta}^T\boldsymbol{\delta}. (10)$$

$$\frac{\partial l(\widehat{\boldsymbol{h}} - \boldsymbol{\delta};\boldsymbol{\theta})}{\partial\theta_i} = \frac{1}{\sigma^2}\left(\frac{\partial\boldsymbol{h}(\boldsymbol{\varphi}(\boldsymbol{t},\boldsymbol{\theta}^*))}{\partial\theta_i}\right)^T\left(\widehat{\boldsymbol{h}} - \boldsymbol{h}(\boldsymbol{\varphi}(\boldsymbol{t},\boldsymbol{\theta}))\right) - \frac{1}{\sigma^2}\boldsymbol{\delta}^T\frac{\partial\boldsymbol{h}(\boldsymbol{\varphi}(\boldsymbol{t},\boldsymbol{\theta}^*))}{\partial\theta_i}, \ (S7)$$

$$\int \mathcal{L}(\widehat{\boldsymbol{h}};\boldsymbol{\theta}^*)\left(-\nabla_{\boldsymbol{\theta}} \log \mathcal{L}(\widehat{\boldsymbol{h}}-\boldsymbol{\delta};\boldsymbol{\theta}^*)^T\right) d\widehat{\boldsymbol{h}}$$

$$= E\left[\frac{1}{\sigma^2}\left(\widehat{\boldsymbol{h}}-\boldsymbol{h}(\boldsymbol{\varphi}(\boldsymbol{t},\boldsymbol{\theta}^*))\right)^T \nabla_{\boldsymbol{\theta}}\boldsymbol{h}(\boldsymbol{\varphi}(\boldsymbol{t},\boldsymbol{\theta}^*))^T - \frac{1}{\sigma^2}\boldsymbol{\delta}^T\nabla_{\boldsymbol{\theta}}\boldsymbol{h}(\boldsymbol{\varphi}(\boldsymbol{t},\boldsymbol{\theta}^*))^T\right]$$

$$= -\frac{1}{\sigma^2}\boldsymbol{\delta}^T\nabla_{\boldsymbol{\theta}}\boldsymbol{h}(\boldsymbol{\varphi}(\boldsymbol{t},\boldsymbol{\theta}^*))^T \quad (S8)$$

The FIM $F(\boldsymbol{\theta}) = \{F(\boldsymbol{\theta})_{ij}\}_{i,j=1,2,\dots,k}$ at the parameter $\boldsymbol{\theta}^*$ is obtained as follows:

$$\frac{\partial l(\widehat{\boldsymbol{h}}-\boldsymbol{\delta};\boldsymbol{\theta})}{\partial\theta_i} = \frac{1}{\sigma^2}\left(\frac{\partial \boldsymbol{h}(\boldsymbol{\varphi}(\boldsymbol{t},\boldsymbol{\theta}))}{\partial\theta_i}\right)^T\left(\widehat{\boldsymbol{h}}-\boldsymbol{h}(\boldsymbol{\varphi}(\boldsymbol{t},\boldsymbol{\theta}))\right) - \frac{1}{\sigma^2}\boldsymbol{\delta}^T\frac{\partial \boldsymbol{h}(\boldsymbol{\varphi}(\boldsymbol{t},\boldsymbol{\theta}))}{\partial\theta_i}, \quad (S9)$$

$$\frac{\partial l(\widehat{\boldsymbol{h}}-\boldsymbol{\delta};\boldsymbol{\theta})}{\partial\theta_j} = \frac{1}{\sigma^2}\left(\frac{\partial \boldsymbol{h}(\boldsymbol{\varphi}(\boldsymbol{t},\boldsymbol{\theta}))}{\partial\theta_j}\right)^T\left(\widehat{\boldsymbol{h}}-\boldsymbol{h}(\boldsymbol{\varphi}(\boldsymbol{t},\boldsymbol{\theta}))\right) - \frac{1}{\sigma^2}\boldsymbol{\delta}^T\frac{\partial \boldsymbol{h}(\boldsymbol{\varphi}(\boldsymbol{t},\boldsymbol{\theta}))}{\partial\theta_j}, \quad (S10)$$

$$E\left(\frac{\partial l(\widehat{\boldsymbol{h}}-\boldsymbol{\delta};\boldsymbol{\theta}^*)}{\partial\theta_i}\frac{\partial l(\widehat{\boldsymbol{h}}-\boldsymbol{\delta};\boldsymbol{\theta}^*)}{\partial\theta_j}\right)$$

$$= \frac{1}{\sigma^4}E\left(\left(\frac{\partial \boldsymbol{h}(\boldsymbol{\varphi}(\boldsymbol{t},\boldsymbol{\theta}^*))}{\partial\theta_i}\right)^T\left(\widehat{\boldsymbol{h}}-\boldsymbol{h}(\boldsymbol{\varphi}(\boldsymbol{t},\boldsymbol{\theta}^*))\right)\left(\frac{\partial \boldsymbol{h}(\boldsymbol{\varphi}(\boldsymbol{t},\boldsymbol{\theta}^*))}{\partial\theta_j}\right)^T\left(\widehat{\boldsymbol{h}}-\boldsymbol{h}(\boldsymbol{\varphi}(\boldsymbol{t},\boldsymbol{\theta}^*))\right)\right)$$

$$-\frac{2}{\sigma^4}E\left(\boldsymbol{\delta}^T\frac{\partial \boldsymbol{h}(\boldsymbol{\varphi}(\boldsymbol{t},\boldsymbol{\theta}^*))}{\partial\theta_i}\left(\frac{\partial \boldsymbol{h}(\boldsymbol{\varphi}(\boldsymbol{t},\boldsymbol{\theta}^*))}{\partial\theta_j}\right)^T\left(\widehat{\boldsymbol{h}}-\boldsymbol{h}(\boldsymbol{\varphi}(\boldsymbol{t},\boldsymbol{\theta}^*))\right)\right)$$

$$+\frac{1}{\sigma^4}\boldsymbol{\delta}^T\frac{\partial \boldsymbol{h}(\boldsymbol{\varphi}(\boldsymbol{t},\boldsymbol{\theta}^*))}{\partial\theta_i}\left(\frac{\partial \boldsymbol{h}(\boldsymbol{\varphi}(\boldsymbol{t},\boldsymbol{\theta}^*))}{\partial\theta_j}\right)^T\boldsymbol{\delta}$$

$$= \frac{1}{\sigma^4}E\left(\left(\frac{\partial \boldsymbol{h}(\boldsymbol{\varphi}(\boldsymbol{t},\boldsymbol{\theta}^*))}{\partial\theta_i}\right)^T\left(\widehat{\boldsymbol{h}}-\boldsymbol{h}(\boldsymbol{\varphi}(\boldsymbol{t},\boldsymbol{\theta}^*))\right)\left(\frac{\partial \boldsymbol{h}(\boldsymbol{\varphi}(\boldsymbol{t},\boldsymbol{\theta}^*))}{\partial\theta_j}\right)^T\left(\widehat{\boldsymbol{h}}-\boldsymbol{h}(\boldsymbol{\varphi}(\boldsymbol{t},\boldsymbol{\theta}^*))\right)\right)$$

$$+\frac{1}{\sigma^4}\left(\frac{\partial \boldsymbol{h}(\boldsymbol{\varphi}(\boldsymbol{t},\boldsymbol{\theta}^*))}{\partial\theta_i}\right)^T\boldsymbol{\delta}\boldsymbol{\delta}^T\frac{\partial \boldsymbol{h}(\boldsymbol{\varphi}(\boldsymbol{t},\boldsymbol{\theta}^*))}{\partial\theta_j}.$$

$$\left(E\left((\widehat{\boldsymbol{h}}-\boldsymbol{h}(\boldsymbol{\varphi}(\boldsymbol{t},\boldsymbol{\theta}^*)))(\widehat{\boldsymbol{h}}-\boldsymbol{h}(\boldsymbol{\varphi}(\boldsymbol{t},\boldsymbol{\theta}^*)))^T\right) = \Sigma\right)$$

$$= \frac{1}{\sigma^2}\left(\frac{\partial \boldsymbol{h}(\boldsymbol{\varphi}(\boldsymbol{t},\boldsymbol{\theta}^*))}{\partial\theta_i}\right)^T\frac{\partial \boldsymbol{h}(\boldsymbol{\varphi}(\boldsymbol{t},\boldsymbol{\theta}^*))}{\partial\theta_j} + \frac{1}{\sigma^4}\left(\frac{\partial \boldsymbol{h}(\boldsymbol{\varphi}(\boldsymbol{t},\boldsymbol{\theta}^*))}{\partial\theta_i}\right)^T\boldsymbol{\delta}\boldsymbol{\delta}^T\frac{\partial \boldsymbol{h}(\boldsymbol{\varphi}(\boldsymbol{t},\boldsymbol{\theta}^*))}{\partial\theta_j} \quad (S11)$$

Hence, we have

$$\int \mathcal{L}(\widehat{\boldsymbol{h}};\boldsymbol{\theta})\left(-(\boldsymbol{\theta}_{\boldsymbol{\delta}}-\boldsymbol{\theta}^*)^T H_{ij}(\log\mathcal{L}(\widehat{\boldsymbol{h}}-\boldsymbol{\delta};\boldsymbol{\theta}^*))(\boldsymbol{\theta}_{\boldsymbol{\delta}}-\boldsymbol{\theta}^*)\right) d\widehat{\boldsymbol{h}}$$

$$= (\boldsymbol{\theta}_{\boldsymbol{\delta}}-\boldsymbol{\theta}^*)^T\left(-E\left(H_{ij}(\log\mathcal{L}(\widehat{\boldsymbol{h}}-\boldsymbol{\delta};\boldsymbol{\theta}^*))\right)\right)(\boldsymbol{\theta}_{\boldsymbol{\delta}}-\boldsymbol{\theta}^*)$$

$$= (\boldsymbol{\theta}_{\boldsymbol{\delta}}-\boldsymbol{\theta}^*)^T\frac{\partial l(\widehat{\boldsymbol{h}}-\boldsymbol{\delta};\boldsymbol{\theta}^*)}{\partial\theta_i}\frac{\partial l(\widehat{\boldsymbol{h}}-\boldsymbol{\delta};\boldsymbol{\theta}^*)}{\partial\theta_j}(\boldsymbol{\theta}_{\boldsymbol{\delta}}-\boldsymbol{\theta}^*)$$

$$=\frac{1}{\sigma^2}(\boldsymbol{\theta_\delta}-\boldsymbol{\theta}^*)^T\left(\frac{\partial \boldsymbol{h}(\boldsymbol{\varphi}(\boldsymbol{t},\boldsymbol{\theta}^*))}{\partial\theta_i}\right)^T\left(\frac{\partial \boldsymbol{h}(\boldsymbol{\varphi}(\boldsymbol{t},\boldsymbol{\theta}^*))}{\partial\theta_j}\right)(\boldsymbol{\theta_\delta}-\boldsymbol{\theta}^*)$$

$$+\frac{1}{\sigma^4}(\boldsymbol{\theta_\delta}-\boldsymbol{\theta}^*)^T\left(\frac{\partial \boldsymbol{h}(\boldsymbol{\varphi}(\boldsymbol{t},\boldsymbol{\theta}^*))}{\partial\theta_i}\right)^T\boldsymbol{\delta}\boldsymbol{\delta}^T\frac{\partial \boldsymbol{h}(\boldsymbol{\varphi}(\boldsymbol{t},\boldsymbol{\theta}^*))}{\partial\theta_j}(\boldsymbol{\theta_\delta}-\boldsymbol{\theta}^*).(S12)$$

Now, we obtain the relative entropy $D\left(\mathcal{L}(\widehat{\boldsymbol{h}};\boldsymbol{\theta}^*):\mathcal{L}(\widehat{\boldsymbol{h}}-\boldsymbol{\delta};\boldsymbol{\theta_\delta})\right)$ as:

$$D\left(\mathcal{L}(\widehat{\boldsymbol{h}};\boldsymbol{\theta}^*):\mathcal{L}(\widehat{\boldsymbol{h}}-\boldsymbol{\delta};\boldsymbol{\theta_\delta})\right)=\frac{1}{2\sigma^2}\boldsymbol{\delta}^T\boldsymbol{\delta}-\frac{1}{\sigma^2}\boldsymbol{\delta}^T\nabla_\theta\boldsymbol{h}(\boldsymbol{\varphi}(\boldsymbol{t},\boldsymbol{\theta}^*))^T(\boldsymbol{\theta_\delta}-\boldsymbol{\theta}^*)$$

$$+\frac{1}{2}(\boldsymbol{\theta_\delta}-\boldsymbol{\theta}^*)^T F_\delta(\boldsymbol{\theta}^*)(\boldsymbol{\theta_\delta}-\boldsymbol{\theta}^*)+o(\|\boldsymbol{\theta_\delta}-\boldsymbol{\theta}^*\|^2),(S13)$$

where $F_\delta(\boldsymbol{\theta}^*)=E\left(\frac{\partial\log\mathcal{L}(\widehat{\boldsymbol{h}}-\boldsymbol{\delta};\boldsymbol{\theta}^*)}{\partial\theta_i}\frac{\partial\log\mathcal{L}(\widehat{\boldsymbol{h}}-\boldsymbol{\delta};\boldsymbol{\theta}^*)}{\partial\theta_j}\right)$ is the FIM at the parameter $\boldsymbol{\theta}^*$ as follows:

$$\{F_\delta(\boldsymbol{\theta}^*)\}_{ij}=\{F(\boldsymbol{\theta}^*)\}_{ij}+\frac{1}{\sigma^4}\left(\frac{\partial \boldsymbol{h}(\boldsymbol{\varphi}(\boldsymbol{t},\boldsymbol{\theta}^*))}{\partial\theta_i}\right)^T\boldsymbol{\delta}\boldsymbol{\delta}^T\frac{\partial \boldsymbol{h}(\boldsymbol{\varphi}(\boldsymbol{t},\boldsymbol{\theta}^*))}{\partial\theta_j},$$

$$\{F(\boldsymbol{\theta}^*)\}_{ij}=\frac{1}{\sigma^2}\left(\frac{\partial \boldsymbol{h}(\boldsymbol{\varphi}(\boldsymbol{t},\boldsymbol{\theta}^*))}{\partial\theta_i}\right)^T\left(\frac{\partial \boldsymbol{h}(\boldsymbol{\varphi}(\boldsymbol{t},\boldsymbol{\theta}^*))}{\partial\theta_j}\right)(S14)$$

Based on the definition of practical identifiability (**Definition 1**) as

$$\lim_{\|\boldsymbol{\delta}\|\to 0}D\left(\mathcal{L}(\widehat{\boldsymbol{h}};\boldsymbol{\theta}^*):\mathcal{L}(\widehat{\boldsymbol{h}}-\boldsymbol{\delta};\boldsymbol{\theta_\delta})\right)=0,\lim_{\|\boldsymbol{\delta}\|\to 0}\|\boldsymbol{\theta_\delta}-\boldsymbol{\theta}^*\|=0,(S15)$$

we have

$$\lim_{\|\boldsymbol{\delta}\|\to 0}D\left(\mathcal{L}(\widehat{\boldsymbol{h}};\boldsymbol{\theta}^*):\mathcal{L}(\widehat{\boldsymbol{h}}-\boldsymbol{\delta};\boldsymbol{\theta_\delta})\right)=\frac{1}{2}(\boldsymbol{\theta}_\delta-\boldsymbol{\theta}^*)^T F(\boldsymbol{\theta}^*)(\boldsymbol{\theta}_\delta-\boldsymbol{\theta}^*)=0.(S16)$$

$\lim_{\|\boldsymbol{\delta}\|\to 0}\|\boldsymbol{\theta}_\delta-\boldsymbol{\theta}^*\|=0$ if and only if $F(\boldsymbol{\theta}^*)$ is invertible. This conclusion can be generalized to cases where $L>1$. □

Proof of Theorem 2

Proof: On the one hand, the necessary condition for $\min_{\boldsymbol{\theta}} l(\widehat{\boldsymbol{h}};\boldsymbol{\theta})$ is obtained as

$$\boldsymbol{S}^T\boldsymbol{S}\boldsymbol{\theta}=\boldsymbol{S}^T\boldsymbol{b}.(S17)$$

For $\forall\theta_i^*, i=1,2,\dots,k$, based on the **Definition 2**, we optimize the conditional loss function as $\min_{\theta_{j,j\neq i}} l(\widehat{\boldsymbol{h}};\boldsymbol{\theta}|\theta_i)$. First, we perform the elementary matrix transformation to the matrix $\boldsymbol{S}$ as $\widehat{\boldsymbol{S}}=\boldsymbol{S}\boldsymbol{P}_{\boldsymbol{i},\boldsymbol{k}}$, where the matrix $\boldsymbol{P}_{\boldsymbol{i},\boldsymbol{k}}=[\boldsymbol{e}_1,\boldsymbol{e}_2,\cdots,\boldsymbol{e}_{i-1},\boldsymbol{e}_k,\boldsymbol{e}_{i+1},\dots,\boldsymbol{e}_{k-1},\boldsymbol{e}_i]$ is the elementary matrix and the vector $\boldsymbol{e}_i$ is unite vector. Next, we decompose the matrix $\widehat{\boldsymbol{S}}$ as $\widehat{\boldsymbol{S}}=[\boldsymbol{A},\boldsymbol{s}_i],\boldsymbol{A}\in$

$\mathbb{R}^{n\times(k-1)}, \boldsymbol{s_i} \in \mathbb{R}^n$ $(n = N * L \geq k)$, $\boldsymbol{s_i}$ is the ith column of matrix $\boldsymbol{S}$. The parameter $\boldsymbol{\theta}$ is rewritten as $\boldsymbol{\theta} = [\widetilde{\boldsymbol{\theta}}; \theta_i], \widetilde{\boldsymbol{\theta}} \in \mathbb{R}^{k-1}$. The conditional loss function $\min_{\theta_{j,j\neq i}} l(\widehat{\boldsymbol{h}}; \boldsymbol{\theta}|\theta_i)$ is rewritten as

$$\min_{\widetilde{\boldsymbol{\theta}}} l(\widehat{\boldsymbol{h}}; \widetilde{\boldsymbol{\theta}}|\theta_i) = \min_{\widetilde{\boldsymbol{\theta}}} \left\|\boldsymbol{A}\widetilde{\boldsymbol{\theta}} + \boldsymbol{s_i}\theta_i - \boldsymbol{b}\right\|_2^2. (S18)$$

Using the condition that $F(\boldsymbol{\theta}^*) = \boldsymbol{S}^T\boldsymbol{S}$ is invertible, the matrix $\hat{F}(\boldsymbol{\theta}^*) = \widehat{\boldsymbol{S}}^T\widehat{\boldsymbol{S}} = \boldsymbol{P_{i,k}}\, F(\boldsymbol{\theta}^*)\boldsymbol{P_{i,k}}$ is also invertible, and the matrix $\boldsymbol{A}$ is full rank of column. Utilizing the necessary condition of least square, the parameter $\widetilde{\boldsymbol{\theta}}$ is given as:

$$\widetilde{\boldsymbol{\theta}} = \boldsymbol{A}^\dagger(\boldsymbol{b} - \boldsymbol{s_i}\theta_i),\ \boldsymbol{A}^\dagger = (\boldsymbol{A}^T\boldsymbol{A})^{-1}\boldsymbol{A}^T. (S19)$$

Substituted into the conditional loss function, we can obtain the equation as

$$l(\widehat{\boldsymbol{h}}; \widetilde{\boldsymbol{\theta}}|\theta_i) = \left\|\boldsymbol{A}\boldsymbol{A}^\dagger\boldsymbol{b} - \boldsymbol{b} + (\boldsymbol{I} - \boldsymbol{A}\boldsymbol{A}^\dagger)\boldsymbol{s_i}\theta_i\right\|_2^2. (S20)$$

Based on the **Definition 2**, the parameter $\theta_i^* \in \boldsymbol{\theta}^*$ is identifiable when $l(\widehat{\boldsymbol{h}}; \boldsymbol{\theta}|\theta_i)$ is convex function. The loss function $l(\widehat{\boldsymbol{h}}; \widetilde{\boldsymbol{\theta}}|\theta_i)$ is convex if and only if $(\boldsymbol{I} - \boldsymbol{A}\boldsymbol{A}^\dagger)\boldsymbol{s_i} \neq \boldsymbol{0}$. Because the matrix $\boldsymbol{A}$ is full rank of column, we have

$$\begin{vmatrix}(\boldsymbol{A}^T\boldsymbol{A})^{-1} & \boldsymbol{0}\\ \boldsymbol{0} & 1\end{vmatrix} \det\left(\hat{F}(\boldsymbol{\theta}^*)\right) = \begin{vmatrix}\boldsymbol{I}_{(k-1)\times(k-1)} & \boldsymbol{A}^\dagger\boldsymbol{s_i}\\ \boldsymbol{s_i}^T\boldsymbol{A} & \boldsymbol{s_i}^T\boldsymbol{s_i}\end{vmatrix} \neq 0, \boldsymbol{s_i}^T(\boldsymbol{s_i} - \boldsymbol{A}\boldsymbol{A}^\dagger\boldsymbol{s_i}) \neq 0.\ (S21)$$

Because the matrix $\boldsymbol{S}$ is full rank of column, $\boldsymbol{s_i}$ is not zero vector. So, we have $(\boldsymbol{s_i} - \boldsymbol{A}\boldsymbol{A}^\dagger\boldsymbol{s_i}) \neq \boldsymbol{0}$.

On the other hand, if $(\boldsymbol{I} - \boldsymbol{A}\boldsymbol{A}^\dagger)\boldsymbol{s_i} \neq \boldsymbol{0}$, the vector $\boldsymbol{s_i}$ is decomposed as:

$$\boldsymbol{s_i} = \boldsymbol{s_i^{(1)}} + \boldsymbol{s_i^{(2)}}, \quad \boldsymbol{s_i^{(1)}} = \boldsymbol{A}\boldsymbol{A}^\dagger\boldsymbol{s_i}, \quad \boldsymbol{s_i^{(2)}} = (\boldsymbol{I} - \boldsymbol{A}\boldsymbol{A}^\dagger)\boldsymbol{s_i}, \quad \boldsymbol{s_i^{(1)}} \perp \boldsymbol{s_i^{(2)}}$$

$$\boldsymbol{s_i}^T(\boldsymbol{s_i} - \boldsymbol{A}\boldsymbol{A}^\dagger\boldsymbol{s_i}) = \left(\boldsymbol{s_i^{(2)}}\right)^T\boldsymbol{s_i^{(2)}} > 0, \begin{vmatrix}(\boldsymbol{A}^T\boldsymbol{A})^{-1} & \boldsymbol{0}\\ \boldsymbol{0} & 1\end{vmatrix} \det\left(\hat{F}(\boldsymbol{\theta}^*)\right) > 0. (S22)$$

So, $F(\boldsymbol{\theta}^*) = \boldsymbol{P_{i,k}^{-1}}\, \hat{F}(\boldsymbol{\theta}^*)\boldsymbol{P_{i,k}^{-1}}$ is invertible where $\boldsymbol{P_{i,k}}$ is the elementary matrix. □

Proof of Theorem 3

Proof: If $\boldsymbol{s_i} \in \mathrm{range}(\boldsymbol{A})$, $\boldsymbol{s_i}$ is able to be linearly expressed using all columns of matrix $\boldsymbol{A}$ as:

$$\boldsymbol{s_i} = \sum_{i=1}^{k-1} \alpha_i\boldsymbol{A}_i\,, \boldsymbol{A} = [\boldsymbol{A}_1, \boldsymbol{A}_2, \dots, \boldsymbol{A}_{k-1}]. (S23)$$

We have the equation as:

$$(\boldsymbol{I} - \boldsymbol{A}\boldsymbol{A}^\dagger)\boldsymbol{s_i} = \sum_{i=1}^{k-1} \alpha_i\boldsymbol{A}_i - \sum_{i=1}^{k-1} \alpha_i\boldsymbol{A}\boldsymbol{A}^\dagger\boldsymbol{A}_i\,. (S24)$$

Because of $\boldsymbol{A}\boldsymbol{A}^\dagger\boldsymbol{A}_i = \boldsymbol{A}_i$, the equation $(\boldsymbol{I} - \boldsymbol{A}\boldsymbol{A}^\dagger)\boldsymbol{s_i} = \boldsymbol{0}$ is obtained.

If the parameter θ_i^* is none-identifiable, it shows $(\boldsymbol{I} - \boldsymbol{A}\boldsymbol{A}^\dagger)\boldsymbol{s_i} = \boldsymbol{0}$ and $\boldsymbol{s_i} \in \mathrm{range}(\boldsymbol{A})$. □

Naturally, the parameter θ_i^* is identifiable if and only if $\boldsymbol{s_i}$ is decomposed as $\boldsymbol{s}_i^{(1)} \in \mathrm{range}(\boldsymbol{A})$ and $\boldsymbol{s}_i^{(2)} \in \ker(\boldsymbol{A}^T)$, and $\boldsymbol{s}_2 \neq \boldsymbol{0}$. As the basic theorem of linear algebra follows:

$$\mathrm{range}(\boldsymbol{A}) = (\ker(\boldsymbol{A}^T))^{\perp}, \dim(\mathrm{range}(\boldsymbol{A})) + \dim(\ker(\boldsymbol{A}^T)) = n, (S25)$$

we have $(\boldsymbol{I} - \boldsymbol{A}\boldsymbol{A}^{\dagger})\boldsymbol{s_i} = \boldsymbol{s}_i^{(2)} \neq \boldsymbol{0}$.

Proof of Theorem 4

Proof: The Eq. (22) is equivalent to an optimization problem as:

$$\min_{\boldsymbol{\theta} \in U(\boldsymbol{\theta}^*, \delta)} \sum_{j=1}^{M} \left\| \boldsymbol{h}\left(\boldsymbol{\varphi}\left(t_{i_j}, \boldsymbol{\theta}\right)\right) - \boldsymbol{h}\left(\boldsymbol{\varphi}\left(t_{i_j}, \boldsymbol{\theta}^*\right)\right) \right\|_2^2 . (S26)$$

If $\boldsymbol{\theta}^*$ is structurally none-identifiable, we can find a parameter $\widehat{\boldsymbol{\theta}} \in U(\boldsymbol{\theta}^*, \delta)$ that satisfies $\boldsymbol{h}\left(\boldsymbol{\varphi}(t, \widehat{\boldsymbol{\theta}})\right) = \boldsymbol{h}(\boldsymbol{\varphi}(t, \boldsymbol{\theta}^*))$, that is, $\forall \left\{t_{i_j}\right\}_{j=1}^{M} \subseteq \{t_i\}_{i=1}^{\infty}$, we have $\boldsymbol{h}\left(\boldsymbol{\varphi}\left(t_{i_j}, \widehat{\boldsymbol{\theta}}\right)\right) = \boldsymbol{h}\left(\boldsymbol{\varphi}\left(t_{i_j}, \boldsymbol{\theta}^*\right)\right), j = 1,2, \ldots, M$. The necessary condition of above optimization problem at the time series $\left\{t_{i_j}\right\}_{j=1}^{M}$ is written as

$$\boldsymbol{s}(\boldsymbol{\theta}^*)^T \boldsymbol{s}(\boldsymbol{\theta}^*)\left(\widehat{\boldsymbol{\theta}} - \boldsymbol{\theta}^*\right) = \boldsymbol{0}. (S27)$$

It demonstrates that $\boldsymbol{s}(\boldsymbol{\theta}^*)^T \boldsymbol{s}(\boldsymbol{\theta}^*)$ is a singular matrix, which is contradicted with the original hypothesis that $\boldsymbol{s}(\boldsymbol{\theta}^*)$ has column full rank. □

Section 2 Supplementary Figures

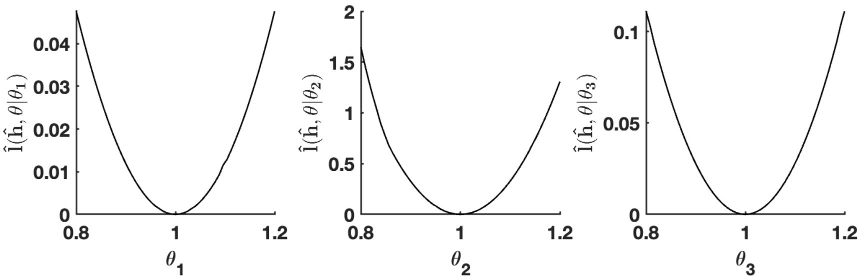

Fig. S1. Practical identifiability analysis of parameters in polynomial fitting with the loss function enhanced by parameter regularization.

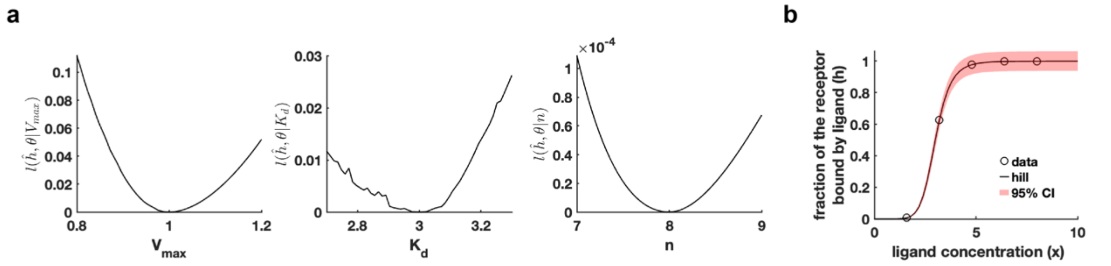

Fig. S2. Practical identifiability analysis of parameters in Hill function fitting. a. Practical identifiability analysis using the profile likelihood. **b.** Uncertainty confidence interval through the perturbation to all parameters. perturbation to all parameter set as 10^{-3}.

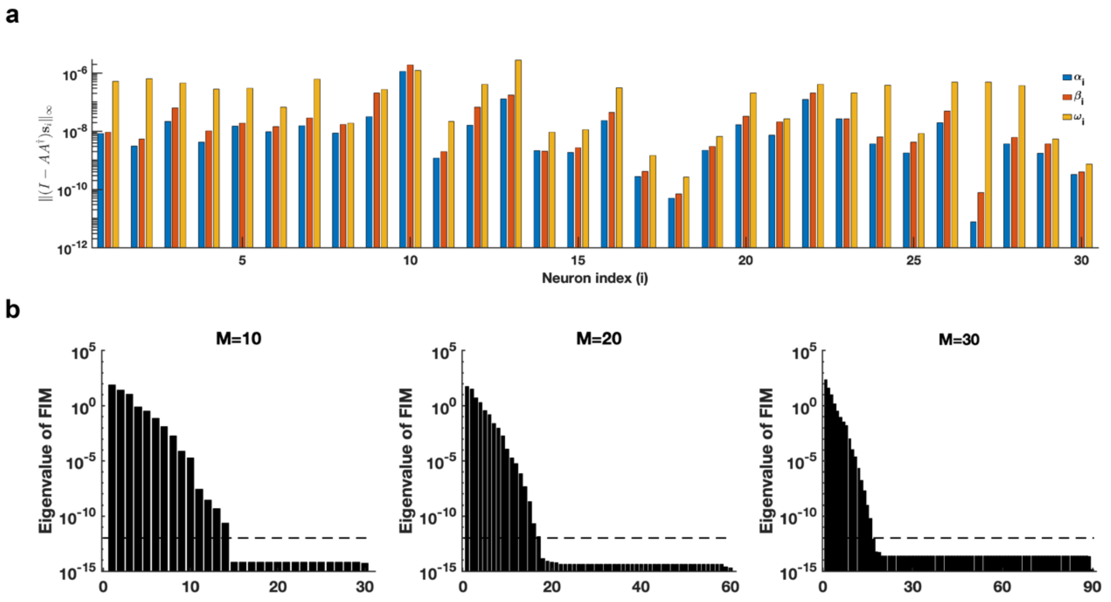

Fig. S3. Practical identifiability analysis of parameters in neural network. (a). Identifiable neurons recognized by the metric $\|(I - AA^{\dagger})s_i\|_\infty$ when the activation function set to tanh function and the number of neurons is assigned as 30. **(b).** Eigenvalue distribution of FIM across different numbers of neurons (M)**.** The dashed line is the threshold $\varepsilon = 10^{-12}$ of eigenvalue of $\boldsymbol{F}(\boldsymbol{\theta}^*)$**.** The activation function set to tanh function.

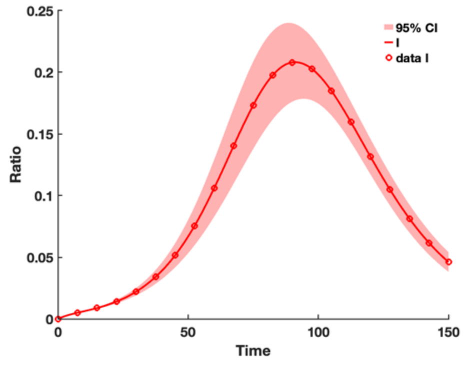

Fig. S4. Practical identifiability analysis of parameters in SEIR model. Uncertainty confidence interval through the perturbation to all parameters using the synthetic data. Perturbation to all parameter set as 10^{-5}.

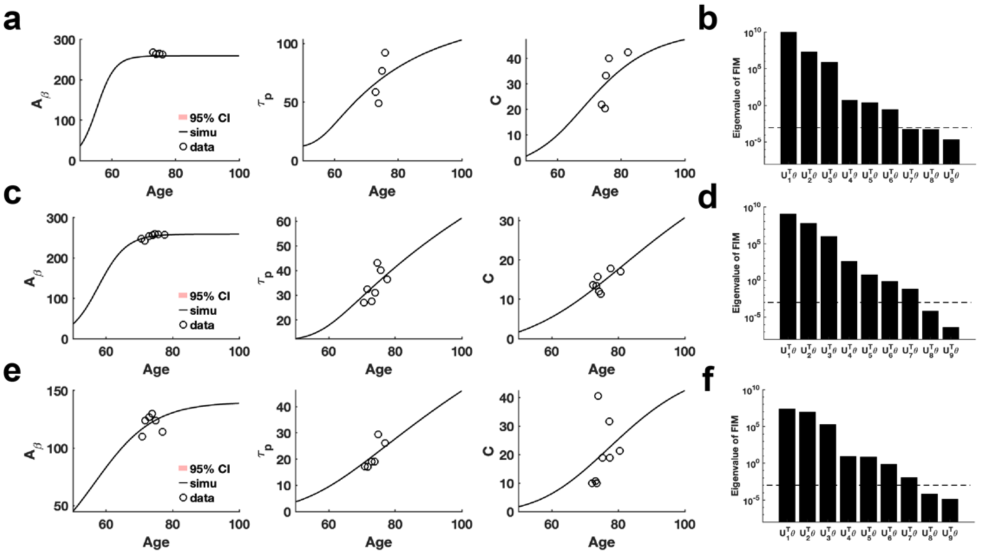

Fig. S5. Practical identifiability analysis of parameters in SEIR model. (a). Uncertainty quantification of AD patient is performed by introducing perturbations to non-identifiable parameters. **(b)** Eigenvalue of AD patients. **(c).** Uncertainty quantification of LMCI patient is performed by introducing perturbations to non-identifiable parameters. **(d)** Eigenvalue of LMCI patients. **(e).** Uncertainty quantification of CN patient is performed by introducing perturbations to non-identifiable parameters. **(f)** Eigenvalue of CN patients. The dash line is the threshold $\varepsilon = 10^{-3}$. Circles represent the real data of AD, LMCI and CN patients. The solid line represents the time course of biomarkers of patients with the given parameter values. The red area represents the 95% confidence interval under parameter perturbation.

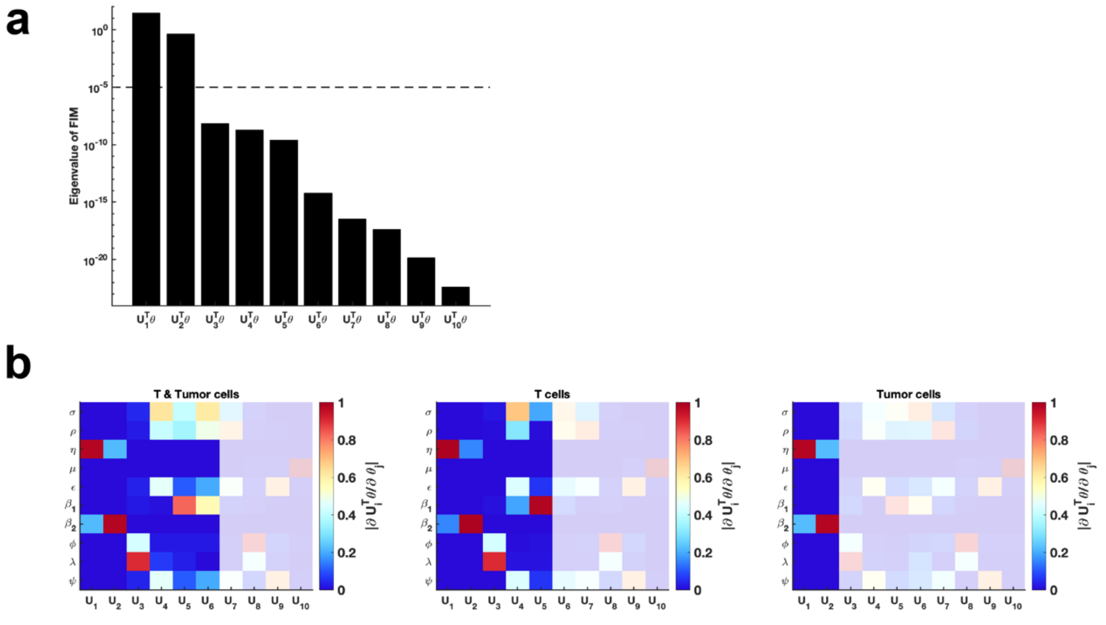

Fig. S6. Practical identifiability analysis of parameters in PDE model of cancer-immune interaction. (a). Eigenvalue of FIM using the glioblastoma data. The dash line is the threshold $\varepsilon = 10^{-5}$. **(b).** Heatmap of the eigenvector matrix in the three cases of observable variables.

Section 3 Values of the parameters

Fig. 2. Perturbation to non-identifiable parameter set as 10, and perturbation to all parameter set as 0.1.

Fig. 3. Perturbation to non-identifiable parameter set as 10. The threshold of eigenvalue for critical data (ε) is set to 1×10^{-5}.

Fig. 4. Perturbation to non-identifiable parameter set as 10^{11}.

Fig. 5. $\boldsymbol{\theta}^* = [0.545,0.028,0.024,0.803]^T$; Perturbation to all parameter set as 10^{-6}; $\boldsymbol{\varphi_0} = [33.956,5.933]^T$ (*3*).

Fig. 6. $\boldsymbol{\theta}^* = [10^6, 10^{-4}, 0.1]^T$ (*4*); Perturbation to non-identifiable parameter set as 0.5; $\boldsymbol{\varphi_0} = [5 \times 10^{-7}, 2 \times 10^{-7}, 0,0]^T$ (*4*). The threshold of eigenvalue for critical data (ε) is set to 5×10^{-5}.

Fig. 7. Synthetic data: $\boldsymbol{\theta}^* = [0.2,0.1,0.06]^T$, $\boldsymbol{\varphi_0} = [0.99,0.01,0,0]^T$ and Perturbation to non-identifiable parameter set as 10^{-2}.
Influenza data: $\boldsymbol{\theta}^* = [5.4, 5.8, 4.54]^T$, $\boldsymbol{\varphi_0} = [1.0,0,1.9 \times 10^{-5}, 0]^T$ and Perturbation to non-identifiable parameter set as 100.

Fig. 8. AD data:
$\boldsymbol{\varphi_0} = [36.03,12,38,0.18,1.68]^T$ (*5*),
$\boldsymbol{\theta}^* = [0.349,0.0182,0.058,0.165,0.041,259.446,123.350,0.9997,50,4803]^T$,
Perturbation to non-identifiable parameter set as 10^{-4};
LMCI data:
$\boldsymbol{\varphi_0} = [36.03,12,38,0.22,1.68]^T$ (*5*)
$\boldsymbol{\theta}^* = [0.237,0.0066,0.01,0.167,0.021,259.440,123.350,1.00,50,480]^T$,
Perturbation to non-identifiable parameter set as 10^{-4}**;**
CN data:
$\boldsymbol{\varphi_0} = [44.92,3.68,0.52,1.68]^T$ (*5*),
$\boldsymbol{\theta}^* = [0.1136,0.0096,0.0196,0.0147,0.077,139.94,123.350,1.00,50,480]^T$,
Perturbation to non-identifiable parameter set as 10^{-4}.

Fig. 9. Glioblastoma data:
$\boldsymbol{\theta}^* = [6.86 \times 10^4, 7.49 \times 10^6, 2.74,2.6 \times 10^6, 3.12 \times 10^7, 1.8 \times 10^5, 0.808,2.57 \times$

$10^5, 2.37 \times 10^6, 3.12 \times 10^7]^T$,

$\boldsymbol{\varphi_0} = [1.92 \times 10^6, 2.0 \times 10^7, 1.92 \times 10^6]^T$ (6)

Perturbation to non-identifiable parameter set as 5×10^8 . The spatial discretization number N is assigned as 128 in the one-dimensional space.

Synthetic data:

$\boldsymbol{\theta}^* = [4.12 \times 10^4, 59960, 0.0404, 6.5 \times 10^7, 3.12 \times 10^7, 1.8 \times 10^5, 1.0, 4.29 \times 10^5, 1.58 \times 10^4, 3.12 \times 10^7]^T$,

Perturbation to non-identifiable parameter set as 3×10^8 . The spatial discretization number N is assigned as 128 in the one-dimensional space.

Section 4 Supplementary Tables

Table S1 Parameter of Cascade model of Alzheimer's Disease (*5*)

Parameters	Biological description
λ_{A_β}	Growth rate of amyloid beta
λ_τ	Growth rate of Tau protein
$\lambda_{N_{\tau_p}}$	Growth rate of Neurodegeneration associated with Tau protein
λ_{CN}	Growth of cognitive decline associated with neurodegeneration
λ_{C_τ}	Growth of cognitive decline associated with Tau protein
K_{A_β}	carrying capacity of amyloid beta
K_{τ_p}	carrying capacity of Tau protein
K_N	carrying capacity of Neurodegeneration
K_C	carrying capacity of cognitive decline

Table S2 Parameter of PDE model of cancer-immune interaction (*6*)

Parameters	Biological description
σ	Normal rate of flow of mature lymphocytes into the tissue
ρ	Proliferation rate of T cells
η	EC50 of tumor cells
μ	positive constant of 'local' kinetic
ϵ	positive constant of 'local' kinetic
β_1	maximal growth rate of the tumor cell
β_2	maximal growth rate of the tumor cell
ϕ	positive constant of 'local' kinetic
λ	positive constant of 'local' kinetic
μ	positive constant of 'local' kinetic
ψ	positive constant of 'local' kinetic
ω	random motility coefficient of the tumor cells

References

1. T. J. Rothenberg, Identification in parametric models. *Econometrica: Journal of the Econometric Society*, 577–591 (1971).
2. C. Kreutz, An easy and efficient approach for testing identifiability. *Bioinformatics* **34**, 1913–1921 (2018).
3. P. Howard, Modeling basics. *Lecture Notes for Math* **442** (2009).
4. D. J. Higham, Modeling and simulating chemical reactions. *SIAM Review* **50**, 347–368 (2008).
5. W. Hao, S. Lenhart, J. R. Petrella, Optimal anti-amyloid-beta therapy for Alzheimer's disease via a personalized mathematical model. *PLoS Comput Biol* **18**, e1010481 (2022).
6. A. Matzavinos, M. A. J. Chaplain, V. A. Kuznetsov, Mathematical modelling of the spatio-temporal response of cytotoxic T-lymphocytes to a solid tumour. *Mathematical Medicine and Biology* **21**, 1–34 (2004).
